# Cell-autonomous and non-cell autonomous effects of neuronal BIN1 loss *in vivo*

**DOI:** 10.1371/journal.pone.0220125

**Published:** 2019-08-13

**Authors:** Kathleen M. McAvoy, Hameetha Rajamohamed Sait, Galina Marsh, Michael Peterson, Taylor L. Reynolds, Jake Gagnon, Sarah Geisler, Prescott Leach, Chris Roberts, Ellen Cahir-McFarland, Richard M. Ransohoff, Andrea Crotti

**Affiliations:** 1 Biogen, Cambridge, MA, United States of America; 2 Department of Cell Biology, Harvard Medical School, Boston, MA, United States of America; University of Modena and Reggio Emilia, ITALY

## Abstract

BIN1 is the most important risk locus for Late Onset Alzheimer’s Disease (LOAD), after ApoE. BIN1 AD-associated SNPs correlate with Tau deposition as well as with brain atrophy. Furthermore, the level of neuronal-specific BIN1 isoform 1 protein is decreased in sporadic AD cases in parallel with neuronal loss, despite an overall increase in BIN1 total mRNA. To address the relationship between reduction of BIN1 and neuronal cell loss in the context of Tau pathology, we knocked-down endogenous murine Bin1 via stereotaxic injection of AAV-Bin1 shRNA in the hippocampus of mice expressing Tau P301S (PS19). We observed a statistically significant reduction in the number of neurons in the hippocampus of mice injected with AAV-Bin1 shRNA in comparison with mice injected with AAV control. To investigate whether neuronal loss is due to deletion of Bin1 selectively in neurons in presence Tau P301S, we bred Bin1^flox/flox^ with Thy1-Cre and subsequently with PS19 mice. Mice lacking neuronal Bin1 and expressing Tau P301S showed increased mortality, without increased neuropathology, when compared to neuronal Bin1 and Tau P301S-expressing mice. The loss of Bin1 isoform 1 resulted in reduced excitability in primary neurons *in vitro*, reduced neuronal c-fos expression as well as in altered microglia transcriptome *in vivo*. Taken together, our data suggest that the contribution of genetic variation in BIN1 locus to AD risk could result from a cell-autonomous reduction of neuronal excitability due to Bin1 decrease, exacerbated by the presence of aggregated Tau, coupled with a non-cell autonomous microglia activation.

## Introduction

Alzheimer’s disease (AD) is the most common form of aging-related dementia currently affecting an estimated 5.7 million Americans (www.alz.org). AD is characterized by cognitive decline associated with accumulation of Amyloid β (Aβ plaques), hyper-phosphorylated misfolded Tau, and neuronal loss [1–3]. Susceptibility loci that contribute to Late Onset of AD (LOAD) have been identified by Genome Wide Association Studies (GWAS). Among them, noncoding Single Nucleotide Polymorphisms (SNPs) associated with the gene expressing *Bridging INtegrator 1* (BIN1) represent the most statistically significant after ApoE (www.Alzgene.org) [4], [5]. Since structural variants of AD-associated BIN1 haven’t been identified yet, the mechanisms underlying the association of genetic variation at the BIN1 locus with AD remain uncertain.

BIN1 is a member of the BIN1/Amphiphysin/RVS167 (BAR) family of adaptor proteins that regulates lipid membrane dynamics[6,7]. *BIN1* is ubiquitously expressed[8], with the highest expression in skeletal muscle[9], followed by brain[8,10]. BIN1 is involved in a wide range of cellular functions including membrane trafficking, clathrin-mediated endocytosis, actin dynamics and apoptosis, depending on cell type [11–16]. *BIN1* is expressed in at least 12 different isoforms generated by alternative splicing[10]. BIN1 isoform 1, exclusively expressed in neurons[17,18], is involved in neurite growth, presynaptic cytoskeleton structural integrity, and fission of synaptic vesicles in neurons[11,12,17,19]. Interestingly, BIN1 -also called Amphishysin II[8]- interacts with Amphiphysin I in neurons[18]. In mice, Amphiphysin I whole body knock-out (KO) leads to loss of Bin1 selectively in neurons[11], suggesting that Amphiphysin I may stabilize Bin1, potentially via heterodimerization. Mice lacking Amphiphysin I and Bin1 in neurons show defects in synaptic vesicle recycling, increased mortality, major learning deficits and higher propensity to seizure[11].

*BIN1* AD-associated SNPs significantly correlate with the level of pTau and total Tau, but not with Αβ in the Cerebrospinal Fluid (CSF) and brains of AD affected individuals [20],[21]. Recently, it has been shown that neuronal BIN1 directly interacts with Tau, likely promoting its deposition [20,22,23]. Furthermore, it has been shown that specific BIN1 AD-associated SNPs correlate with reduced entorhinal and temporal pole cortical thickness as assessed by neuroimaging[24] as well as atrophy of hippocampus, CA1 and parahippocampus[21]. Also, BIN1 SNP rs744373 risk allele is associated with worse high-load working memory performance and lower functional connectivity between the bilateral hippocampi and the right dorsolateral prefrontal cortex[25].

BIN1 AD-associated SNPs are located in the intergenic regulatory region upstream of the *BIN1* Transcription Start Site (TSS), raising the question whether BIN1 expression levels are affected by AD-associated risk alleles[20,26]. It has been reported that the level of neuronal-expressed BIN1 isoform 1 protein, measured by WB, was decreased in AD post-mortem brain samples[27,28]. Further, decreased neuronal BIN1 paralleled the decrease of several neuronal markers, as observed by Immunofluorescence (IF)[29,30]. One interpretation of these data was that BIN1 reduction varied directly with neuronal loss, because the protein was reduced as a consequence of the cell loss. Another possibility suggested by the genetic relationship between BIN1 AD-associated SNPs and brain atrophy is that the loss of neuronal Bin1 was causally linked to neuronal loss. Here, we show that acute knock-down of total Bin1 in adulthood via stereotaxic injection of AAV-shRNA leads to neuronal loss in the hippocampus of Tau P301S (PS19) mice. Surprisingly, genetic ablation of Bin1 specifically in post-mitotic excitatory neurons leads to increased mortality, but does not affect neuronal number in PS19 background. Interestingly, loss of Bin1 isoform 1 reduced neuronal excitability *in vitro* as well as modulating neuronal activation and microglia transcriptome *in vivo*. Our observations suggest that reduced neuronal BIN1 could contribute to the progression of AD by cell-autonomous reduction of neuronal activity, as well as non-cell autonomous microglia activation.

## Material and methods

### Ethical statement

All experimental procedures involving animals were approved by the Institutional Animal Care and Use Committee at Biogen, and every effort was made to minimize suffering. All procedures involving animals were performed in accordance with standards established in the Guide for the Care and Use of Laboratory Animals as adopted by the U.S. National Institutes of Health. Biogen is accredited by the Association for Assessment and Accreditation of Laboratory Animal Care (AAALAC) International.

### Adeno-Associated viruses

AAV (1/8)-GFP-U6-scrmb-shRNA (cat: 7040), AAV (1/8)-GFP-U6-mBin1-shRNA (cat: shAAV253986), AAV (1/8)-GFP-U6-rBin1-shRNA (cat: shAAV-293232), were prepared and CsCl-purified by Vector Biolabs (https://www.vectorbiolabs.com).

### Rat primary cortical neurons preparation and infection

Pregnant rats were sacrificed at gestational day E16-18. Embryos heads were removed and placed in HBSS (Cat: 14025092, Thermo Fisher) and brain harvested. Hippocampus and cortex were dissected and placed in a 10cm dish filled with fresh HBSS and treated with Trypsin (Cat: T4549, Sigma) for 20 min at 37°C in gentle rotation. Subsequently, tissue was treated with DNAse I (Cat:DN25, Sigma), dissociated by pipetting, washed with 10ml DMEM/10% FBS and spun for 5min at 1,200rpm. Pellet was resuspended in Neurobasal media (Cat: 21103, Gibco), GlutaMax (Cat: 35050, Gibco), and N21 supplement (Cat: SCM081, Millipore) and 1% Pen/Strep (Cat: 15140–122, Gibco). After trituration and strain through 100μm cell strainer, neurons were counted and plated at 35,000 cells/well in Corning BioCoat Poly-D-Lysine 96 Multiwell Plate (Cat:08-774-27, Corning). On Days In Vitro (DIV) 7 or DIV14, neurons were infected with AAV (1/8)-GFP-U6-rBin1-shRNA (titer 2.6 x10^13^ GC/ml) or AAV (1/8)-GFP-U6-scrmb-shRNA (titer 5.3 x10^13^ GC/ml). Amount of each virus used to infect neurons had been normalized based on viral titer. Neurons received one-third media replacement at DIV4, 10. At DIV14, half media had been replaced with Brain Phys B-P media (cat: 05790, Stem Cell) containing N21, P/S and glucose (2.5μM). Rat neuronal cultures were healthy based on phase-contrast imaging of cell bodies (bright, with visible nucleus) and dendrites (no beading or blebbing) and maintained adherence to the substrate for up to 3 weeks. On the day of assay, media was replaced with Brain Phys B-P media containing 20μM glycine. FLIPR Assay had been performed using FLIPR Calcium 6 Assay Kit according to manufacturer’s instructions (cat: R8190, Molecular Devices). Measurement was performed with in FlexStation® instruments.

### Hippocampal organotypic brain slices culture preparation and infection

Brains were dissected from decapitated postnatal p2-p4 C57BL/6J pups (Cat: 000664, The Jackson Laboratories) and glued (Glue Loctite) to the chuck of a water-cooled vibratome (Leica VT1000A) and trimmed with a commercial shave razor. Under aseptic conditions, 300μm-thick coronal sections were sliced and collected in sterile medium. The organotypic slices were then placed in a 0.4-μm membrane insert (Millipore, PICM03050) in a 6-well plate and cultured in presence of Basal Medium containing HBSS 1X, 15% Horse serum, GlutaMax, 45% Glucose, Pen-strep, N2 supplement, and fresh PDGF (10ng/ml) and maintained at 35°C and 5% CO_2_. Slices were infected with the respective AAV after 3 days of culturing. After AAV infection, slices were cultured for 9–12 days, during which time half of the media volume was replaced with one half fresh media every three days. Slices were collected and lysed with Lysis Buffer as described below.

### Western blots

Rat Primary Cortical Neurons, Hippocampal Organotypic Brain Slices, dissected Cortexes or Hippocampi were lysed in Lysis Buffer (140mM NaCl, 10mM HEPES pH7.4, 1% NP-40) plus 1mM PMSF, 1mM DTT, Complete Protease Inhibitor Cocktail (Cat:11836170001, Roche). After 1h incubation on ice, lysates were cleared by spinning at 13,000rpm for 10min at 4°C. Protein concentrations were determined by BCA protein assay (Cat: 23228, Thermo Scientific). One hundred micrograms of protein per sample were mixed with LDS buffer (Cat: B0007, Life Technologies), heated at 70°C for 10min and run on NuPage Tris-Acetate 7% gel using Tris-Acetate SDS Running Buffer (Cat log: LA0041, Life Technologies). All the gels were run at 120V for 2h and then transferred to a Nitrocellulose membrane (iblot 1B23001, Life Technologies) with iblot-2 (Thermo Scientific). After 1h blocking in 5% (w/v) non-fat milk in TBS with 0.1% Tween 20, the membranes were then incubated overnight at 4°C with the following antibodies diluted 1:500 in TBS with 0.1% Tween 20: Bin1 99D (Cat: sc-13575, Santa Cruz), Bin1 (Ab153912, Abcam), GAPDH 1:2000 (Ab189095, Abcam), Tubulin (Cat: MAB1195, R&D). The membranes were washed in TBST (4×10 min) before 1h incubation with anti-rabbit (Cat: GTX213110-01, Genetex) or anti- mouse (Cat: sc-2055, Santa Cruz) conjugated HRP at 1:1000 dilution. The immunoblots were then visualized using ECL (Cat: 32209, Thermo Scientific). Images were acquired with Amersham Imager 600 (GE Healthcare Bio-Sciences, PA) with time of exposure ranging between 10sec. and 10min., at a resolution of 600dpi. For densitometry analysis, the image files were opened in ImageJ and converted to 8 bit Grey scale. Bands were selected using the Rectangular selection tool, with the same region of interest in all the lanes. Each gel was analyzed using the plot lane, yielding a histogram-based intensity of the exposure and background. Using the straight line tool, a straight line was drawn at the base of the each peak, the peaks highlighted with the wand tool, and analyzed with the label peaks function. The measurements of Area and Percentage area analyzed were normalized with controls.

### Mouse lines

Mouse strains B6.129S6-Bin1tm2Gcp/J (Bin1flox/flox), Thy1-Cre <FVB/N-Tg(Thy1-cre)1Vln/J> (Thy1-Cre) and B6;C3-Tg(Prnp-MAPT*P301S)PS19Vle/J (PS19) were purchased from Jackson Laboratories (cat. No. 021145, 006143 and 008169 respectively). Mice were housed with a 12h light/dark cycle, and food and water were provided *ad libitum*.

### Behavior tests

#### Open field

To measure general locomotor activity, mice were assessed in the open field task. Prior to the task, mice were acclimated to the testing room for 30min. They were then placed in an opaque cylindrical bucket 40 cm in diameter. During the 30min session, an overhead camera captured animal activity. After testing, mice were returned to their home cage, and the buckets cleaned with Quatricide. Data was analyzed by the Noldus Ethovision program to obtain the total distance traveled during the session.

#### Cued and contextual fear conditioning

On day 1 of training, mice were placed into Coulbourn Habitest conditioning chambers (7"Wx7"Dx12"H) for a total of 9 minutes. The session consists of 2 CS-US pairings (30sec white noise, 2 sec 0.5mA foot shock). The mice were given 180 sec of free exploration before the first CS-US pairing and had 150 seconds between the subsequent CS-US pairing. Mice remained in the chamber for 150 seconds after the last CS-US pairing. Twenty-four hours after training, mice were returned to the conditioning chamber for contextual fear testing (10 minutes) and freezing behavior which was assessed by FreezeFrame2 software. Approximately 2 hours after contextual testing, conditioning chambers were altered by the addition of white plastic floors/walls that were distinct from training context. Altered context freezing was assessed for 3 minutes prior to exposure to the white noise CS (Cued testing), which lasted for 6 minutes. Freezeframe2 captured the freezing scores throughout. Boxes were thoroughly wiped with Quatricide prior to the first mouse, between each mouse, and after the last mouse. Quatricide was allowed to thoroughly evaporate prior to placing animals in chambers. Animals were 6 months of age at time of testing.

#### Startle and pre-pulse inhibition

Animals were placed in a small holding chamber that is mounted atop a motion-sensitive platform all enclosed in a foam-lined acoustic cubicle that measures 25”x16.5”x15.5”. Speakers were located directly behind the platform and there was a light and a fan inside the cubicle to provide lighting and ventilation. Mice were pseudo-randomly exposed to pre-pulses (subthreshold for producing a startle response), pulses (producing a maximal startle response), or pre-pulses (55, 59, 63, or 67dB) 100ms before pulses (110dB). Session contained 48 trials, which lasts 20 minutes total. Med-Associates software captured the startle response in terms of latency to react and the magnitude of startle reaction. Pre-Pulse Inhibition (PPI) was calculated for each pre-pulse intensity using the following formula:

$$\frac{100\;x\;(S–PPi\_S)}{S}$$

S is the average response to the startle alone and PPi is the average response to the startle immediately preceded by a pre-pulse. Boxes were thoroughly wiped with Quatricide prior to the first mouse, between each mouse, and after the last mouse. Quatricide was allowed to thoroughly evaporate prior to placing animals in chambers.

#### Pentylenetetrazole (PTZ) seizure testing

On the day of testing, Pentylenetetrazole (Cat: P6500, Sigma-Aldrich) was prepared in sterile saline at 10 mg/mL and drawn into a 1 mL BD syringe attached to a tail vein catheter (ReCathCo), consisting of a 29 gauge needle with a 600 mm PE tubing. The syringe from the catheter was then placed in an infusion pump (KAI infusions). Prior to injection, mice were placed in a heated incubator set at 37°C for 5 minutes for the purpose of dilating the tail vein. Mice were then restrained in a mouse restrainer. The tail was wiped with an alcohol pad and the lateral tail vein located. The needle from the tail vein catheter was inserted into the lateral tail vein and placement confirmed with the presence of blood entering the catheter. Immediately after placement, PTZ was injected at a constant rate of 0.250 ml/min. The time latencies from the start of the infusion to seizure, noted as the initial tremor, or “First Jerk” reaction were recorded. Infusion is immediately stopped at the appearance of tonic forelimb and/or hindlimb extension (i.e. “Mortality”) and the time recorded. The seizure threshold dose was calculated by:

$$\frac{rate\;of\;infusion\;(\frac{ml}{min})*time\;of\;onset\;(s)*concentration\;PTZ\;(\frac{mg}{ml})*1000}{60*body\;weight\;of\;animal\;(g)}$$

### AAV stereotaxic injection in PS19 mice

Male PS19 mice at 3 months of age received a single stereotaxic injection of 1.5μl of AAV(1/8)-GFP-U6-mBin1-shRNA (3.6x10^13^ GC/ml) or AAV(1/8)-GFP-U6-scrmb-shRNA (5.3x10^13^ GC/ml) at hippocampal coordinates: bregma antero-posterior 2.5 mm, medial-lateral -1.7 mm, and dorso-ventral 1.6 mm, following the procedure described below. Mice were given Buprenorphine Animalgesics (sustained release, 3.25mg/kg s.c.) and anesthetitization with Isoflurane (first in a chamber (3% mixed with oxygen) and then maintained with a nose cone (1.25% maintenance). After mice were immobilized in a stereotaxic apparatus, an incision was made over bregma, and a burr hole was drilled through the skull. The study reagent was injected using a motorized microinjector and a 10μl gas-tight Hamilton microsyringe at rate of 0.5μl/min. The needle was allowed to remain for 2–4 minutes following injection to prevent reflux of fluid and then slowly removed over 2 minutes. After needle removal, the skin incision was closed with wound glue. Mice were monitored daily for a minimum of 7 days to ensure proper recovery and absence of infection. Starting at 6 months of age, mice were weighed and monitored weekly, following guidelines reported below. When reaching Clinical score 2, the mouse was euthanized, and intracardially perfused with saline solution. The brain was collected and fixed in Neutral Formaline Buffer (Cat: 5701, Thermo Fisher).

### PS19 monitoring

Mice expressing Tau P301S (PS19) show onset of symptoms around 6 months of age [31]. Thus, we implemented a system of monitoring based on the following Clinical Scoring (CS): CS 0 Normal; CS 1 Abnormal gait (low walk with good mobility in cage), able access food and water and/or body weight loss (BWL) ≈10%; CS 2 Mild hind limb weakness/paresis effecting both or one leg, hind limb muscle wastage noted. Good mobility in cage and/or 10%<BWL≤20%; CS 3 Moderate hind limb paresis with or without front limb weakness/paresis, able to right on both left and right side within 15 seconds when placed lateral recumbent and/or 20%<BWL<32%. Fair mobility in cage; CS4 Severe hind limb paresis with or without front limb weakness/paresis, not able to right when placed lateral recumbent on either the right or left side, and/or paralysis in any limbs and/or body weight loss ≥32%. Poor mobility in cage.

### Immunohistochemistry

#### Tissue preparation

Mice were cardiovascularly perfused with PBS and brains were fixed in 10% neutral-buffered formalin for 72–96 hours (Cat: 032–059, Fisher Scientific). The right side of each brain was marked by ink. Brains were dissected coronally into 3 or 6 slabs, processed and blocked on a Tissue Tek VIP tissue processor (Sakura Finetek) and embedded into paraffin in an anterior face down orientation. Five-micron thick paraffin sections were generated either manually (using a Leica rotary microtome RM2255) or on an automated slide preparation instrument AS-400 (Dainippon Seiki, Japan). Serial 5-micron sections were collected from each sample and placed on charged slides. For each sample, 2 or 3 sections, separated by approximately 200–250 micron, were subjected to immunohistochemistry analysis.

#### Immunohistochemistry (IHC)

IHC staining was performed on an automated Ventana Discovery Ultra platform. Briefly, slides were baked at 60°C and deparaffinized. Heat-mediated epitope retrieval was performed in the Ventana CC1 retrieval solution (pH 8.0) and then slides were incubated with 3%H_2_O_2_ to quench the endogenous peroxidase activity. Slides were incubated with respective primary antibody solutions for 60min, followed by secondary antibodies if applicable. Thereafter, slides were incubated with polymer horseradish peroxidase-conjugated antibodies (Ventana, Cat: 760–4205, or rabbit polyclonal anti-chicken IgG, Cat: 303-005-003, Jackson Labs), followed by addition of DAB. The following antibodies were used: chicken polyclonal anti-GFP (Cat: Ab13970, Abcam, final concentration 2μg/ml), rabbit monoclonal anti-NeuN (clone D4G4O, Cell Signaling Technology, Cat. No. 24307, final concentration 0.26 μg/mL), rabbit polyclonal anti-Iba1 (Wako Chemicals, Cat. No. 019–19741, final concentration 0.25 μg/mL). Slides were counterstained with hematoxylin, dehydrated and mounted.

#### Immunofluorescence (IF)

For immunofluorescence labeling of AAV infected cell types, deparaffinized hippocampal sections prepared as above were incubated with primary antibodies overnight at 4°C in PBS with 0.3% Triton-X and 10% Normal Donkey Serum or Normal Goat Serum (Jackson Immunoresearch). After three PBS washes, sections were incubated for 90 minutes with species-specific cross-absorbed secondary antibodies (Jackson Immunoresearch, Cat: 111-547-008, 111-167-008, 706-165-148), washed in PBS, and coverslipped with Fluoromount containing DAPI (Southern Biotech). Antibodies included: chicken polyclonal anti-GFP (Cat: Ab13970, Abcam, final concentration 10μg/ml), rabbit polyclonal anti-Iba1 (Cat: 019–19741,Wako, final dilution 1:1000), rabbit polyclonal anti-GFAP (cat:NB300-141, Novus, final dilution 1:1000), rabbit polyclonal anti-NeuN (cat: Ab104225, Abcam, final concentration 1μg/ml), guinea pig polyclonal anti-NeuN (cat: ABN90, Millipore, final dilution 1:1000), mouse monoclonal anti-Gad67 clone 1G10.2 (cat: MAB5406, Millipore, final dilution 1:1000). For immunofluorescence analysis of c-fos (guinea pig polyclonal anti- c-fos, cat: 226–004, Synaptic Systems, final dilution 1:1000) and Iba1 (rabbit polyclonal anti- Iba1, Cat: 019–19741, Wako, final dilution 1:1000; number of microglia cells S6 Fig) hippocampal cryo-sections prepared from paraformaldehyde (PFA) and saline perfused mice were incubated as above. For the ‘Novel Environment Exposure’ exposure, mice were placed in a circular area (diameter 40cm) with opaque walls in which a small amount (~30g) of bedding from a cage of unfamiliar female mice had been placed in the center. Mice were allowed to explore the area for 30 minutes, after which they were returned to their home cage for one hour prior to perfusion.

#### Imaging and analysis

Stained slides were scanned at 20x magnification on a 3DHistech Panoramic P250 Slide scanner. Embedding pathology marker dye was used to confirm correct left/right orientation on digital images. Images were analyzed with custom image analysis algorithms using Visiopharm image analysis software. Images were manually annotated by blinded analyst bilaterally on all slides. Annotated regions (hippocampus and entorhinal Cortex) were analyzed based on Visiopharm’s Decision Forest classifier to detect DAB as NeuN, Iba1 or GFP indirect immunostaining. Relative area of NeuN, Iba1 or GFP immunostaining was calculated as a percent of total tissue area in annotated regions. The Iba1 microglia count was performed by detecting cell soma, identified by punctate Iba1 immunostaining, gated on size. Microglial dendritic process were only the Iba1 immunostained regions contiguous to the cell soma. Area measurements were a direct product of the algorithm per tissue region area. For immunofluorescence analysis of AAV infected cell types in hippocampal organotypic slices, tiled full thickness 20x images of the entire hippocampus were obtained on a Zeiss Axio Observer using ZEN software. Overlap between GFP-expressing cells and GFAP, NeuN, Gad67 or Iba1 cell-type markers was manually scored by a blinded observer. For immunofluorescence analysis of c-fos and Iba1 number of microglia cells, 20x Z-stack images through the entire section depth were captured of select hippocampal and cortical regions. Images were captured and maximum intensity projections generated using Slidebook software. Cells were manually counted by an investigator blind to experimental condition.

### Microglia sorting

#### Microglia isolation

Briefly, animals were anesthetized with Ketamine/Xylazine i.p. injection (1:1, concentration) and ice-cold PBS was used for perfusion and brains collection. After removing Cerebellum and Olfactory bulbs, remaining brain tissue was minced in Petri dish and transferred to 15ml tube with 3ml of Accutase (Cat: SCR005, Millipore); and incubated at 4°C for 30min in a rotary shaker. Then, large tissue chunks were allowed to settle at the bottom by gravity, and the cell suspension from the top of the tube were passed through pierce tissue strainer (Cat: 87791, Thermo Fisher) and collected in fresh tubes. Two ml of HBSS (Cat: 14185–052, Life Technologies)/HEPES (Cat: 15630–080, Life Technologies) buffer was added to the chunk tissues and triturated with 5ml pipette until almost homogenous. Following gravity separation, supernatants were passed through a pierce tissue strainer and pooled to the same tube containing cell suspension from previous step. Trituration was repeated with the remaining tissue chunks with a 1ml pipette until completely homogenous and passed through a pierce strainer and pooled to the rest of the cell suspension. Then, the strainer was rinsed with HBSS/HEPES buffer to bring the cell suspension to 15ml, prior to a spin at 600xg for 5min at 4°C (brake 5). The supernatant was aspirated, and the pellet was resuspended with 1ml of 100% FBS. Nine milliliters of 33% Percoll (Cat: 17089102, GE Life Science) was added to the cell suspension and mixed well. One milliliter of 10%FBS was overlayed on the Percoll/cell suspension and spun at 800g at 4°C for 15min (break 1 or lowest possible). The supernatant was aspirated without disturbing the pellet, and the pellet was resuspended with 1ml of FACS buffer (HBSS, 1%BSA, 2mM EDTA, 25mM HEPES, 0.09% NaN_3_). Additional FACS buffer was added to bring the cell suspension to 15mL before spinning at 600g for 10min at 4°C. Following centrifugation, the pellet was resuspended with 300μl of FACS buffer and filtered through 30μm filter (Partec Celltrics, 04-004-2326) for further analysis.

#### Cell staining for sorting

Filtered cells were pelleted down with gentle centrifugation and incubated with 50μl of Fc blocker anti-mouse CD16/32 (Cat: 101321, Biolegend) on ice for 10min. Then 50μl of CD11b-PE Rat anti-mouse antibody (Cat: 553311, BD Bioscience) and CD45 BV421 anti-mouse antibody (Cat: 563890, BD Bioscience) were mixed with the cells and incubated for 30min on ice. Following incubation, cells were washed and resuspended in 200μl of FACS buffer. Cells were filtered through a 30μm filter before FACS sorting (BD FACS Aria Fusion) and collecting approximately 20,000–100,000 CD11b^+^; CD45^med^ cells in FACS buffer. Unstained and single-stained cells were used as controls for gating the cells in FACS. Sorted cells were centrifuged at 6800g for 15min and the cell pellets were snap frozen and stored at -80°C until RNA extraction.

### RNAseq

#### RNA extraction

Qiagen All Prep kit (Cat: 80224, Qiagen) was used for RNA extraction from previously frozen microglia cells. Briefly, cells were lysed with RLT Buffer (with β-mercaptoethanol added), and passed through QIA shredder (Cat: 79654, Qiagen). Flow-through from the QIA shredder was then passed through a DNA Mini column to remove the DNA from the lysate. DNA mini columns were stored at 4°C for DNA purification later; and the passed-through lysates were treated with proteinase K. Following treatment, 100% ethanol was added to the lysates and passed through an RNeasy Mini spin column. In-column DNase treatment was also performed. Subsequently, RNA was eluted with RNase-free water, quantified in Nano-drop and stored at -70°C until samples were submitted for sequencing.

#### RNA sequencing

cDNA from each sample was generated from 10ng of total RNA using SMARTer Ultra Low Input RNA kit v4 (Clontech). The cDNA was amplified by 8 PCR cycles, followed by QC analysis on BioAnalyzer 2100 (Agilent). Sequence libraries were produced from 150pg of cDNA using Nextera XT DNA library kit (Illumina), cleaned up with AMPure XP beads, and QC checked with Caliper LabChip GX. Single-end sequencing data were generated on an Illumina HiSeq 2500, at a depth of 30 million reads per sample, with read length 50bp.

#### RNASeq analysis

The reads were aligned with the OmicSoft OSA4 to the mouse genome (mm10) and the Ensembl.R84 gene model. Gene counts were estimated using RSEM. Two samples were removed (one sample from WT_Female cohort and one samples from Heterozygote_Male cohort) due to low fraction of uniquely mapped single reads and higher than expected duplication. Of note, these samples with poor technical QC metrics were also clear outliers in PCA. Normalization and differential expression analysis were carried out with the Bioconductor package DESeq2. Differentially expressed genes (DEGs) were defined as having adjpval < 0.05, and |FC| > 1.5. The lists of DEGs were analyzed for pathway and ontology enrichment using the Ingenuity IPA software.

#### Accession code

GSE135230

### Statistical methods

Statistical analyses were performed using R’s linear mixed modeling function, lme. The fixed effect portion models 2-way effects with interaction. The random effect portion employed random intercepts per animal and within-subject correlations were modeled. Residual diagnostic plots assessed the assumptions of the linear mixed model and log transformation was applied when needed. Some models employed un-equal variance mixed modeling via the weights argument of lme. P-values were adjusted for multiplicity using the glht single-step method. Time to event/survival analysis was performed for Bin1fl/fl; Thy1-Cre; PS19 mice for the time to clinical score 2, the time to clinical score 3, and survival as measured by the survival alt variable for each gender. Kaplan-Meier Survival Curves were calculated for each scenario comparing wild-type, heterozygous, and knock out mice. Pairwise logrank tests were conducted for heterozygous vs wild-type mice and knock out versus wild type mice for each scenario and log rank p-values were adjusted for multiplicity using Holm’s method. The assumption of proportional hazards was verified with a proportional hazards test (cox.zph function in R) as well as visual inspection of the cloglog diagnostic plot. Lastly, the median time to event (in days) was calculated for each scenario for each group. No outliers were identified or excluded. Adjusted p-values less than 0.05 were considered significant. Additional statistical analyses were performed using GraphPad Prism Two-way ANOVA with Tukey’s multiple comparison test. D’Agostino & Pearson’s omnibus normality tests had shown that the distribution of the values in most of the experiment is normal. No outliers were identified or excluded. All experiments were performed as a minimum of 3 independent replicates. Adjusted p-values less than 0.05 were considered significant.

## Results

### Acute knock-down of Bin1 in the hippocampus of PS19 mice results in neuronal loss

**Fig 1:**
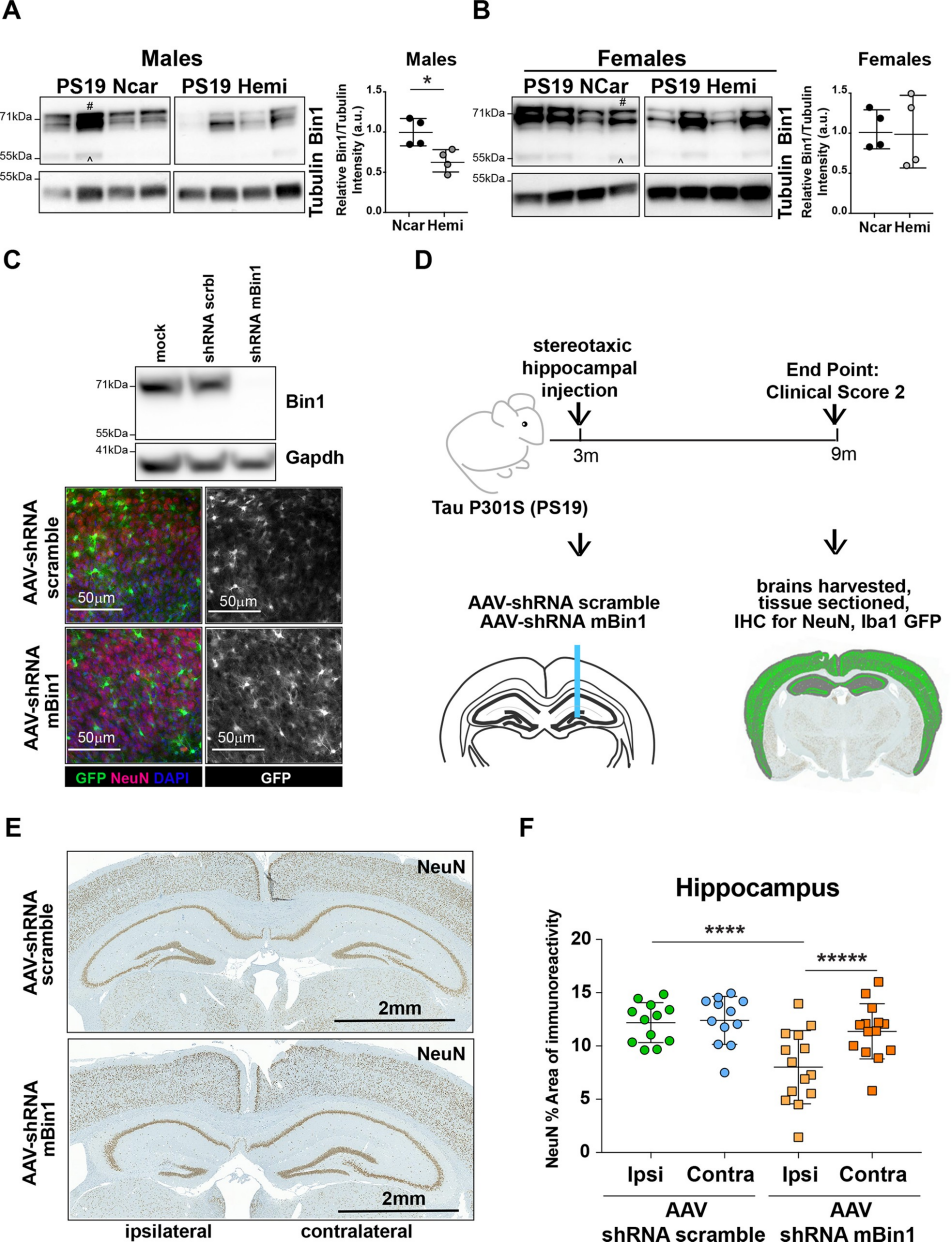
Acute knock-down of *Bin1* in the hippocampus of PS19^Hemi^ mice results in neuronal loss. **(A) (B)** Western blotting of brain lysates from male and female PS19^Ncar^ and PS19^Hemi^ mice showing Bin1 expression. Tubulin represents loading control. # indicates neuronal Bin1 isoform 1. ^ indicates ubiquitous Bin1 isoform 2. Plots show Bin1 levels normalized to tubulin in male and female mice, respectively. Each circle represents one animal. Unpaired Student t test: p-value: * p< 0.05; ** <0.01; *** p<0.001. (**C**) Top: western blot shows Bin1 expression in lysates from hippocampal organotypic slice cultures infected with AAV (1/8)-GFP-shRNA scrbl and AAV (1/8)-GFP-shRNA mBin1 on DIV 3 and harvested at DIV 9. Gadph represents loading control. Bottom: immunofluorescence images of hippocampal slice cultures infected with AAV (1/8)-GFP-shRNA scrbl and AAV (1/8)-GFP-shRNA mBin1 and immunostained for NeuN (neuronal marker) as well as GFP (infected cells) and DAPI (nuclear marker). Scale bar: 50μm. One representative image per condition. (**D**) Schematic and timeline of AAV injection paradigm. Inset image shows NeuN stained coronal section, with gray region highlighting total area annotated for neuropathology while green highlights NeuN staining in these regions. (**E**) Immunohistochemical staining of NeuN in hippocampi of PS19^Hemi^ mice injected with AAV (1/8)-GFP-U6-shRNA scrbl and AAV (1/8)-GFP-U6-shRNA mBin1, respectively. One representative image per group. Scale bar: 2mm. (**F**) Plot representing the percent area of NeuN immunoreactivity in the ipsilateral and contralateral hippocampus (area of NeuN immunoreactivity normalized to the total area of the ipsilateral or contralateral hippocampus, respectively, for each animal). Circles represent animals injected with AAV (1/8)-GFP-U6-shRNA scrbl: green filled circles represent ipsilateral hemisphere; blue filled circles represent contralateral hemisphere. Squares represent animals injected with AAV (1/8)-GFP-U6-shRNA mBin1: yellow filled squares represent ipsilateral hemisphere; red filled squares represent contralateral hemisphere. Each circle/square represents one single animal. A linear mixed effect model with a 2-way fixed effect model with interaction, a random intercept, and a compound symmetric within-subject correlation structure was employed. Post-hoc comparisons were analyzed between ipsilateral and contralateral hemispheres within animals, as well as between ipsilateral hemispheres across groups and contralateral hemispheres across groups. P-values were adjusted via the glht single step method: Adjusted p-value: * p< 0.05; ** <0.01; *** p<0.001; **** p<0.0001; ***** p<0.00001.

Due to the correlation between BIN1 AD-associated SNPs and Tau pathology, we sought to determine whether Tau pathology can drive alterations in Bin1 expression. We analyzed the levels of the neuronal isoform of Bin1 in human Tau P301S-expressing mice (PS19^Hemi^) and non-carrier littermates (PS19^Ncar^)[31]. The neuronal-specific Bin1 isoform 1 had been detected as a doublet due to phosphorylation (Fig 1A and 1B), as previously reported in rat as well as in murine brain lysates[15,32]. Interestingly, few samples showed additional Bin1 immunoreactive bands. Nevertheless, the brains of male PS19^Hemi^ mice showed a statistically significant reduction of neuronal Bin1 in comparison to male PS19^Ncar^ mice, at the onset of disease signs (8–9 months of age), whereas female mice, which have a slower onset of pathology[33], did not show a significant reduction (Fig 1A and 1B). These data suggest that Bin1 is reduced at a time point in which Tau aggregates are present and neuronal loss is first noted[31].

Whether reduction of BIN1 contributes to or is the result of AD pathology in human subjects is unknown. Accordingly, we sought to determine whether acute knock-down of Bin1, via AAV expressing shRNA, affects neuropathology in adult PS19 mice *in vivo*. Our preliminary validation showed that AAV(1/8)-GFP-U6-mBin1-shRNA drove substantial knock-down of Bin1 expression in hippocampal organotypic brain slices, and that control AAV(1/8)-GFP-U6-scrmb-shRNA and AAV(1/8)-GFP-U6-mBin1-shRNA infection resulted in similar distribution of GFP immunoreactivity **(**Fig 1C**)**. Male PS19^Hemi^ mice received unilateral stereotaxic injection in hippocampal area of either AAV at 3 months of age, before the onset of Tau pathology, and were sacrificed upon reaching Clinical Score 2 (Methods), when brains were harvested, fixed, sectioned and stained for GFP (to mark AAV-expressing cells), the neuronal marker NeuN (to assess neuropathology) and the microglial marker Iba1 (Fig 1D). No difference was observed in the time required to reach clinical score 2 between the two groups, ranging between 250 and 300 days. As determined by GFP and NeuN, Gad67, GFAP or Iba1 co-staining, AAV (1/8)-GFP-U6-scrmb-shRNA transduced mainly neurons (80% of total transduced cells, a quarter of which were inhibitory neurons (Gad67^+^)), but also astrocytes, and a low number of microglia cells *in vivo* (S1A and S1B Fig). Strikingly, AAV(1/8)-GFP-U6-mBin1-shRNA injected animals showed virtually no remaining shRNA-expressing GFP^+^ cells at the time of brains collection (S1C and S1D Fig). This observation prompted us to investigate whether knock-down of Bin1 could result in neuronal loss *in vivo*. We found a statistically significant reduction in the percent area of NeuN immunoreactivity in the ipsilateral hippocampus of mice injected with AAV expressing mBin1 shRNA in comparison to the ipsilateral hippocampus of mice injected with AAV expressing scramble shRNA (Fig 1E and 1F), consistent with the hypothesis that BIN1 could be implicated in AD pathology-associated neuronal loss. Of note, we observed spreading of control and test AAVs from ipsilateral to contralateral hemispheres in the brains of the injected mice. Interestingly, a statistically significant difference in the percent area of NeuN immunoreactivity was observed between ipsilateral and contralateral hemispheres of brains of mice injected with AAV expressing mBin1 shRNA, suggesting a dose-dependent effect of Bin1 knockdown on neuronal loss (Fig 1F). There was no difference in the total area of the hippocampus itself for AAV(1/8)-GFP-shRNA scrbl or AAV(1/8)-GFP-shRNA mBin1 injected mice, although there was a significant difference in microglial number and microglial area between ipsilateral and contralateral hippocampi of brains of mice injected with AAV expressing mBin1 shRNA, and a significant difference in microglial processes between ipsilateral and contralateral hippocampi of brains of mice injected with AAV(1/8)-GFP-shRNA scrbl (as determined by Iba1^+^ staining) (S2A–S2E Fig). These data suggest that acute knock-down of total Bin1 in the context of human Tau P301S expression results in hippocampal neuronal loss, with subtle effects on microglia number and morphology occurring in injected hemispheres of both control and experimental mice.

### Genetic deletion of Bin1 in neurons leads to a reduction in survival but not to neuronal loss in PS19 Hemi or Ncar mice

**Fig 2:**
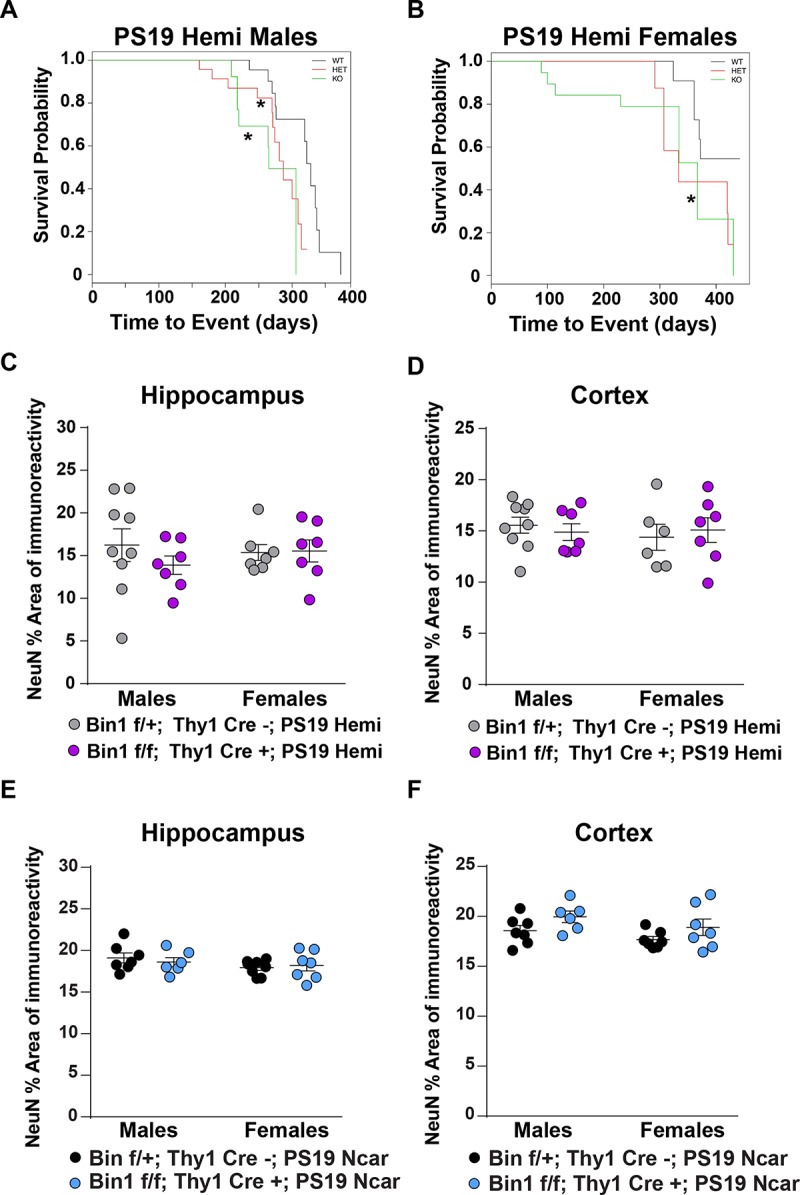
Genetic deletion of Bin1 in neurons leads to a reduction in survival but not to neuronal loss in PS19 Hemi or Ncar mice. (**A**) Male *Bin1*^*flox/flox*^:: *Thy1-Cre*^*+*^:: *PS19*^*Hemi*^ mice (KO, green line) and *Bin1*^*flox/+*^::*Thy1-Cre*^*+*^::*PS19*^*Hemi*^ (HET, red line) show reduced survival probability relative to *Bin1*^*flox/+*^::*Thy1-Cre*^*-*^::*PS19*^*Hemi*^ (WT, black line) mice. Mean time to death WT: 328 days, HET: 287 days (p = 0.029), KO: 265 days (p = 0.029) (Kaplan Meier adjusted p-value (adj), relative to WT). Adjusted p-value: * p< 0.05; ** <0.01; *** p<0.001. (**B**) Female *Bin1*^*flox/flox*^:: *Thy1-Cre*^*+*^:: *PS19*^*Hemi*^ mice (KO, green line) show reduced survival probability relative to *Bin1*^*flox/+*^::*Thy1-Cre*^*-*^::*PS19*^*Hemi*^ (WT, black line) mice. Mean time to death WT: not calculated as greater than 50% remained alive at experimental end point, HET: 333 days (p = 0.097), KO: 367 days (p = 0.017) (Kaplan Meier adjusted p-value, relative to WT). Adjusted p-value: * p< 0.05; ** <0.01; *** p<0.001. **(C)** Plot representing the percentage of NeuN staining area in sections from the hippocampus of male and female *Bin1*^*flox/+*^::*Thy1-Cre*^*-*^::*PS19*^*Hemi*^ (gray filled circles) or *Bin1*^*flox/flox*^:: *Thy1-Cre*^*+*^:: *PS19*^*Hemi*^ mice (purple filled circles) mice, expressed as a percentage of the total hippocampal area. Each circle represents the value from one animal. (**D**) Plot representing the percent area of NeuN immunoreactivity in sections from the cortex of male and female *Bin1*^*flox/+*^:: *Thy1-Cre*^*-*^::*PS19*^*Hemi*^ (gray filled circles) or *Bin1*^*flox/flox*^:: *Thy1-Cre*^*+*^:: *PS19*^*Hemi*^ mice (purple filled circles) mice, expressed as a percentage of the total cortical area. Each circle represents the value from one animal. (**E**) Plot representing the percent area of NeuN immunoreactivity in sections from the hippocampus of male and female *Bin1*^*flox/+*^:: *Thy1-Cre*^*-*^::*PS19*^*Ncar*^ (black filled circles) or *Bin1*^*flox/flox*^:: *Thy1-Cre*^*+*^:: *PS19*^*Ncar*^ mice (blue filled circles) mice, expressed as a percentage of the total hippocampal area. Each circle represents the value from one animal. (**F**) Plot representing the percent area of NeuN immunoreactivity in sections from the cortex of male and female *Bin1*^*flox/+*^:: *Thy1-Cre*^*-*^::*PS19*^*Ncar*^ (black filled circles) or *Bin1*^*flox/flox*^:: *Thy1-Cre*^*+*^:: *PS19*^*Ncar*^ mice (blue filled circles) mice, expressed as a percentage of the total cortical area. Each circle represents the value from one animal. *PS19*^*Ncar*^ mice age: 12–14 months old. *PS19*^*Hemi*^ mice age: 8–9 month-old. As the *PS19*^*Hemi*^ mice and the *PS19*^*Ncar*^ mice were sampled at different ages, due to high mortality in the *PS19*^*Hemi*^ line, no statistical comparisons were made between these two genotypes. Instead, the effect of Bin1 genotype was analyzed within genders within each PS19 genotype, as well as any interactions between Bin1 genotype and gender. (**C**) (**D**) (**E**) (**F**) 2-way ANOVA with interaction with single-step corrected p-values. Results presented in C, D, E and F don’t show statistically significant difference.

The AAV-mediated Bin1 knockdown occurred in excitatory and inhibitory neurons as well as astrocytes (S1A and S1B Fig). We sought to determine whether selective genetic deletion of Bin1 in excitatory neurons affects the development of AD-like pathology in PS19 mice[31]. Since BIN1 AD-associated SNPs correlate with various morphometric measurements of different brain areas of AD-affected individuals[21],[24],[25], we chose to be agnostic with regard to region and ablate Bin1 expression throughout the forebrain. In addition, to avoid potential developmental effects of Bin1 deletion, we bred *Bin1*^*flox/flox*^ with *Thy1-Cre* expressing mice to achieve post-natal deletion of Bin1 gene specifically in forebrain excitatory neurons[34–37]. The expression of the neuronal, but not the ubiquitous, isoform of Bin1 was reduced in these animals (S3A and S6A Figs). We then crossed *Bin1*^*flox/flox*^::*Thy1-Cre* mice (*Bin1-cKO*) onto the PS19 line, achieving a comparable reduction of Bin1 expression (S3B Fig). Interestingly, we observed the appearance of additional Bin1 immunoreactive bands in *Bin1*^*flox/flox*^::*Thy1-Cre*:: *PS19*^*Hemi*^ mice (S3B Fig) that we didn’t observe in *Bin1*^*flox/flox*^:: *Thy1-Cre* mouse line (S3A Fig). Bin1 is subject to phosphorylation[15,32], and such modification regulates its interaction with Tau[22]. It is possible that additional Bin1 immunoreactive bands represent differentially phosphorylated Bin1 proteins. Thus, it is plausible that the concomitant expression of human Tau P301S could affect Bin1 phosphorylation status. Cohorts of *Bin1*^*flox/flox*^:: *Thy1-Cre*^*+*^:: *PS19*^*Hemi*^ (KO), *Bin1*^*flox/+*^::*Thy1-Cre*^*+*^::*PS19*^*Hemi*^ (HET) and *Bin1*^*flox/+*^::*Thy1-Cre*^*-*^::*PS19*^*Hemi*^ (WT) mice were monitored throughout lifespan and scored according a set of clinical parameters (Methods). Male and female homozygous *Bin1-cKO; PS19*^*Hemi*^ mice showed a statistically significant reduction in lifespan (Fig 2A and 2B) and an earlier onset of clinical signs (S2C–S2F Fig). No difference was observed in survival between homozygous or heterozygous *Bin1-cKO* and *Bin1-WT* mice, in absence of Tau P301S overexpression.

We assessed whether post-natal Bin1 deletion in excitatory forebrain neurons induced neuropathology in PS19^Ncar^ mice, or if this deletion increased neuropathology in presence of human Tau P301S *in vivo*. Furthermore, we investigated whether genetic neuronal Bin1 deletion modified cognitive defects observed in PS19^Hemi^ mice, or whether genetic neuronal Bin1 deletion alone resulted in cognitive effects. We measured hippocampal and cortical area (mm^2^), and hippocampal and cortical NeuN area of immunoreactivity (mm^2^), as well as the percentage of NeuN area of immunoreactivity (% area immunoreactive for NeuN) in the following 4 genotypes: *Bin1*^*flox/flox*^:: *Thy1-Cre*^*+*^:: *PS19*^*Hemi*^, *Bin1*^*flox/+*^::*Thy1-Cre*^*-*^::*PS19*^*Hemi*^, *Bin1*^*flox/flox*^:: *Thy1-Cre*^*+*^:: *PS19*^*Ncar*^, *Bin1*^*flox/+*^::*Thy1-Cre*^*-*^::*PS19* ^*Ncar*^. Unexpectedly, we did not observe reduction of neuronal number or total area in hippocampus or cortex (% area NeuN, total area, Area NeuN) in mice lacking Bin1 regardless the expression of human Tau P301S (Fig 2C–2F and S2A–S2H Fig).

We then assessed cognitive impairment in the following 4 genotypes: *Bin1*^*flox/flox*^:: *Thy1-Cre*^*+*^:: *PS19*^*Hemi*^, *Bin1*^*flox/flox*^::*Thy1-Cre*^*-*^::*PS19*^*Hemi*^, *Bin1*^*flox/flox*^:: *Thy1-Cre*^*+*^:: *PS19*^*Ncar*^, *Bin1*^*flox/flox*^::*Thy1-Cre*^*-*^::*PS19*^*Ncar*^. *Bin1*^*flox/flox*^:: *Thy1-Cre*^*+*^::*PS19*^*Ncar*^ mice showed a statistically significant reduction of the total distance traveled in the Open Field test (S5A Fig) at 6 months of age relative to *Bin1*^*flox/flox*^::*Thy1-Cre*^*-*^::*PS19*^*Ncar*^ mice, although it should be noted that on a c57Bl6 background, *Bin1*^*flox/flox*^::*Thy1-Cre*^*+*^ did not show differences in activity compared to *Bin1*^*flox/flox*^::*Thy1-Cre*^*-*^ littermates (*Bin1*^*flox/flox*^::*Thy1-Cre*^*-*^ mean distance 75.58m ± 20.05m; *Bin1*^*flox/flox*^::*Thy1-Cre*^*+*^ mean distance 88.44m ± 20.61m; Student’s t-test not significant). Relative to *Bin1*^*flox/flox*^::*Thy1-Cre*^*-*^::*PS19*^*Ncar*^ mice, *Bin1*^*flox/flox*^:: *Thy1-Cre*^*+*^::*PS19*^*Ncar*^ and *Bin1*^*flox/flox*^:: *Thy1-Cre*^*+*^:: *PS19*^*Hemi*^ mice showed a statistically significant reduced auditory startle, a stereotyped measure of sensorimotor reflex reaction, at 7 months of age (S5B Fig). *Bin1*^*flox/flox*^::*Thy1-Cre*^*+*^:: *PS19*^*Hemi*^ mice showed a statistically significant reduction in paired-pulse inhibition (PPI) at 7 months of age (S5C Fig). Pre-pulse inhibition (PPI) of the auditory startle reflex is considered a measure of forebrain circuitry, which provides a filtering mechanism to allocate attention to salient stimuli. The described deficits in both sensorimotor reflex, and forebrain gating of this reflex, suggest alterations in both cortical and subcortical circuitry[38]. Finally, we assessed hippocampal-independent and hippocampal-dependent memory using cued and contextual fear conditioning. No significant deficit in freezing behavior was detected in *Bin1*^*flox/flox*^:: *Thy1-Cre*^*+*^::*PS19*^*Ncar*^ or *Bin1*^*flox/flox*^:: *Thy1-Cre*^*+*^:: *PS19*^*Hemi*^ mice, regardless of sex and genotypes combinations (S5D and S5E Fig). These data suggest that loss of Bin1 in forebrain excitatory neurons produces subtle alterations in cognitive function and warrants further exploration with additional behavioral assays.

### Genetic deletion of Bin1 in excitatory neurons alters neuronal activation *in vivo*

**Fig 3:**
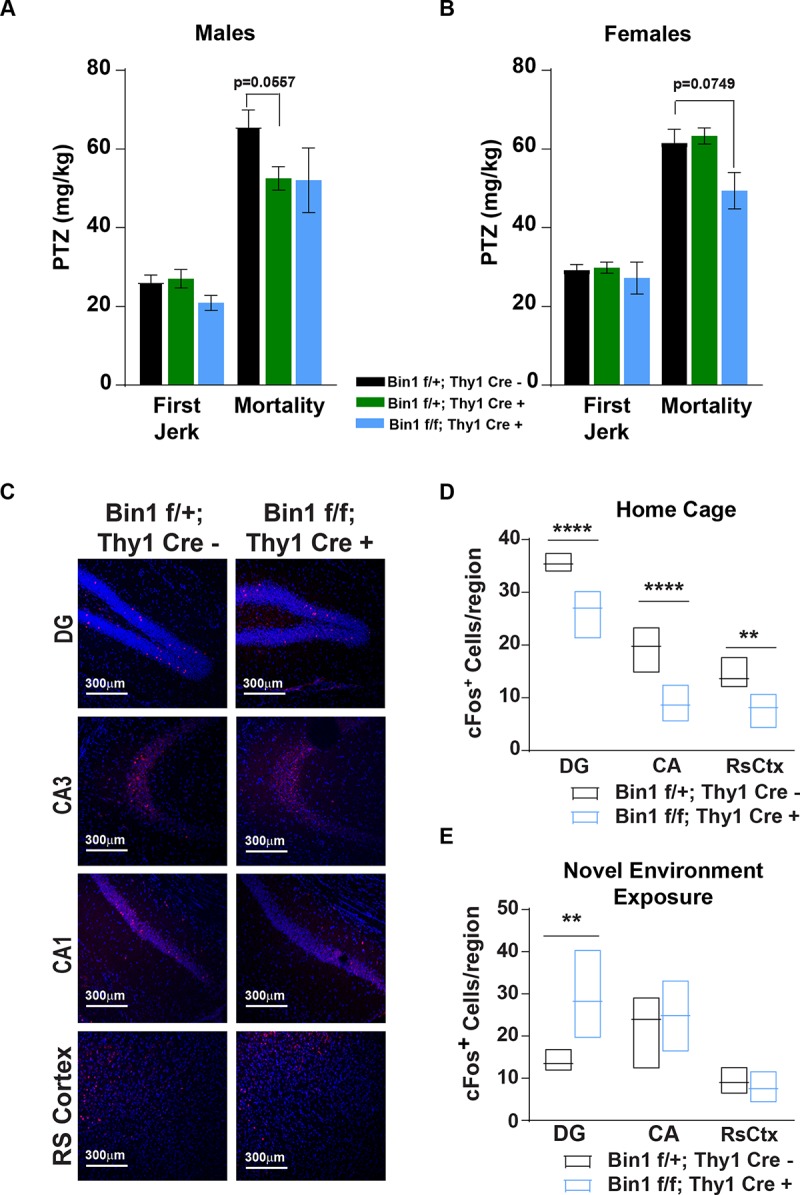
Genetic deletion of Bin1 in neurons alters neuronal activation *in vivo*. **(A) (B)** Plots representing PTZ doses required to elicit seizure activity (”First Jerk” and”Mortality”) in *Bin1*^*flox/flox*^:: *Thy1-Cre*^*+*^ (blue bars, N = 4 males, 8 females); *Bin1*^*flox/+*^::*Thy1-Cre*^*+*^ (green bars, N = 13 males, 13 females) and *Bin1*^*flox/+*^::*Thy1-Cre*^*-*^ (black bars, N = 14 males, 16 females) male and female mice, respectively. Data are shown as mean **±** SEM of 2 independent experiments. A linear mixed effect model with a 2-way fixed effect model with interaction, a random intercept, and a compound symmetric within-subject correlation structure was employed. P-values were adjusted via the glht single step method. No significant differences identified, p-values indicated for clarity. (**C**) Representative immunofluorescence images of c-fos labeling in select brain regions from *Bin1*^*flox/+*^:: *Thy1-Cre*^*-*^ and *Bin1*^*flox/flox*^:: *Thy1-Cre*^*+*^ mice sacrificed directly from their Home Cage. DAPI (blue). c-fos (red). Scale bar: 300μm. (**D**) (**E**) Plots representing the number of c-fos^+^ cells per region in animals sacrificed from their “Home Cage” or after”Novel Environment Exposure”, respectively. *Bin1*^*flox/+*^:: *Thy1-Cre*^*-*^ (black bars) and *Bin1*^*flox/flox*^:: *Thy1-Cre*^*+*^ (blue bars). Boxes represent range, with line representing mean. N = 4 mice per group. A linear mixed effect model was employed which included: 2-way fixed effects with interaction, a random intercept, and within-subject correlations. P-values were adjusted via the glht single step method. Adjusted p-value: * p< 0.05; ** <0.01; *** p<0.001; **** p<0.0001.

**Fig 4:**
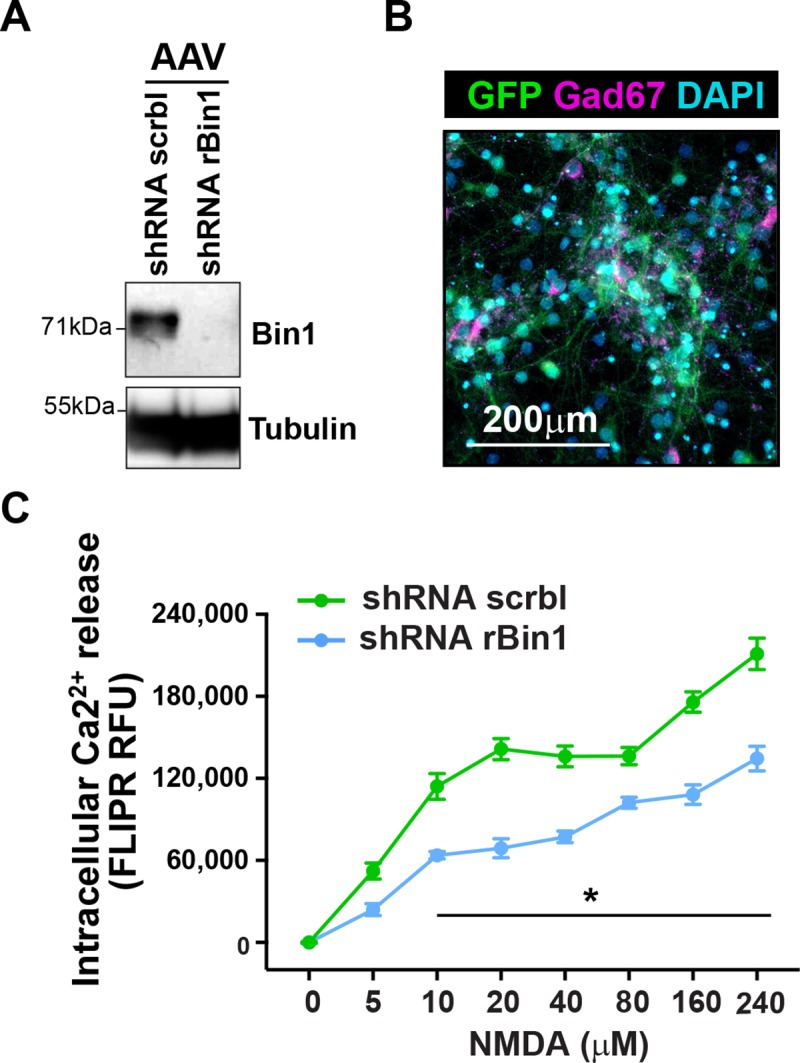
Acute knockdown of Bin1 reduces neuronal activity *in vitro*. **(A)** Western blot image showing Bin1 levels in lysates from cultured rat cortical neurons infected with AAV-GFP-U6-shRNA-scramble (control) and AAV-GFP-U6-shRNA-rBin1 (shRNA targeting rat Bin1). Representative image from three independent biological replicates. **(B)** Representative image of cultured rat cortical neurons on DIV 14 stained for GFP, Gad67 (inhibitory cell marker) and DAPI. Scale bar: 200μm. (**C**) Plot representing intracellular Ca2^+^ release, as measured by FLIPR Relative Fluorescence Unit (RFU), in cultured rat cortical neurons infected with AAV-GFP-U6-shRNA-scramble and AAV-GFP-U6-shRNA-rBin1, respectively. FLIPR RFU represents the mean area under curve for fluorescence readings post-NMDA addition. Plot shows one representative experiment out of four independent biological replicates. Each point represents the mean **±** SD of 6 wells per condition. Two-way ANOVA with Tukey’s multiple comparison test: p-value: * p< 0.05; ** <0.01; *** p<0.001.

Given that genetic deletion of Bin1 in forebrain excitatory neurons, in the presence of Tau P301S expression, results in decreased animal survival without worsening neuropathology, and that the same deletion has subtle effects on behavior, we sought to broadly assess the functional role of Bin1 in the context of circuit activation, using *Bin1*^*flox/flox*^:: *Thy1-Cre*^*+*^ and littermate controls. To this end, we assessed seizure susceptibility using Pentylenetetrazole (PTZ), infused via tail vein injection, leading to rapidly rising CNS PTZ concentrations and allowing assessment of both threshold dose for first clonus and death due to continuous tonic-clonic convulsions[39]. No statistically significant reduction in the dose of PTZ necessary to induce "Mortality” or to trigger the “First Jerk” was identified regardless of sex (Fig 3A and 3B). We then analyzed the effect of neuronal Bin1 deletion on hippocampal neuronal activation, monitored with c-fos immunoreactivity (IR)[40]. Male mice were sacrificed directly from the “Home Cage” environment (HC) or following a “Novel Environment Exposure”, and brains harvested and stained for c-fos. Deletion of neuronal Bin1 resulted in reduced hippocampal c-fos^+^ neurons in dentate gyrus (DG), CA, and retrosplenial cortex in non-stimulated mice, sacrificed directly from HC (Fig 3C and 3D). Conversely, loss of neuronal Bin1 resulted in increased c-fos^+^ neurons exclusively in the DG of mice exposed to a novel environment (Fig 3E). To directly assess neuronal excitability in the context of Bin1 loss, we utilized primary rat cortical neurons in which Bin1 expression had been knocked-down by AAV-GFP-U6-rBin1-shRNA. This shRNA targeting rat Bin1 infected the majority of neurons, including inhibitory neurons (Gad-67 expressing) and reduced Bin1 expression (Fig 4A and 4B). Since the induction of c-fos transcription, and subsequent detectable IR c-fos+ neurons, occurs following neuronal activation through sensitivity to intracellular calcium concentration changes[41,42], we assessed intracellular calcium release in primary cortical neurons lacking Bin1 expression after NMDA stimulation. Loss of Bin1 expression resulted in a dose-dependent, statistically significant decreased intracellular calcium release in response to multiple NMDA doses (Fig 4C). Analysis of c-fos *in vivo* and NMDA-mediated calcium release *in vitro* as proxy measures of neuronal activation suggest that Bin1 plays a functional role to promote neuronal activity.

### Loss of Bin1 in neurons affects microglia gene expression *in vivo*

**Fig 5:**
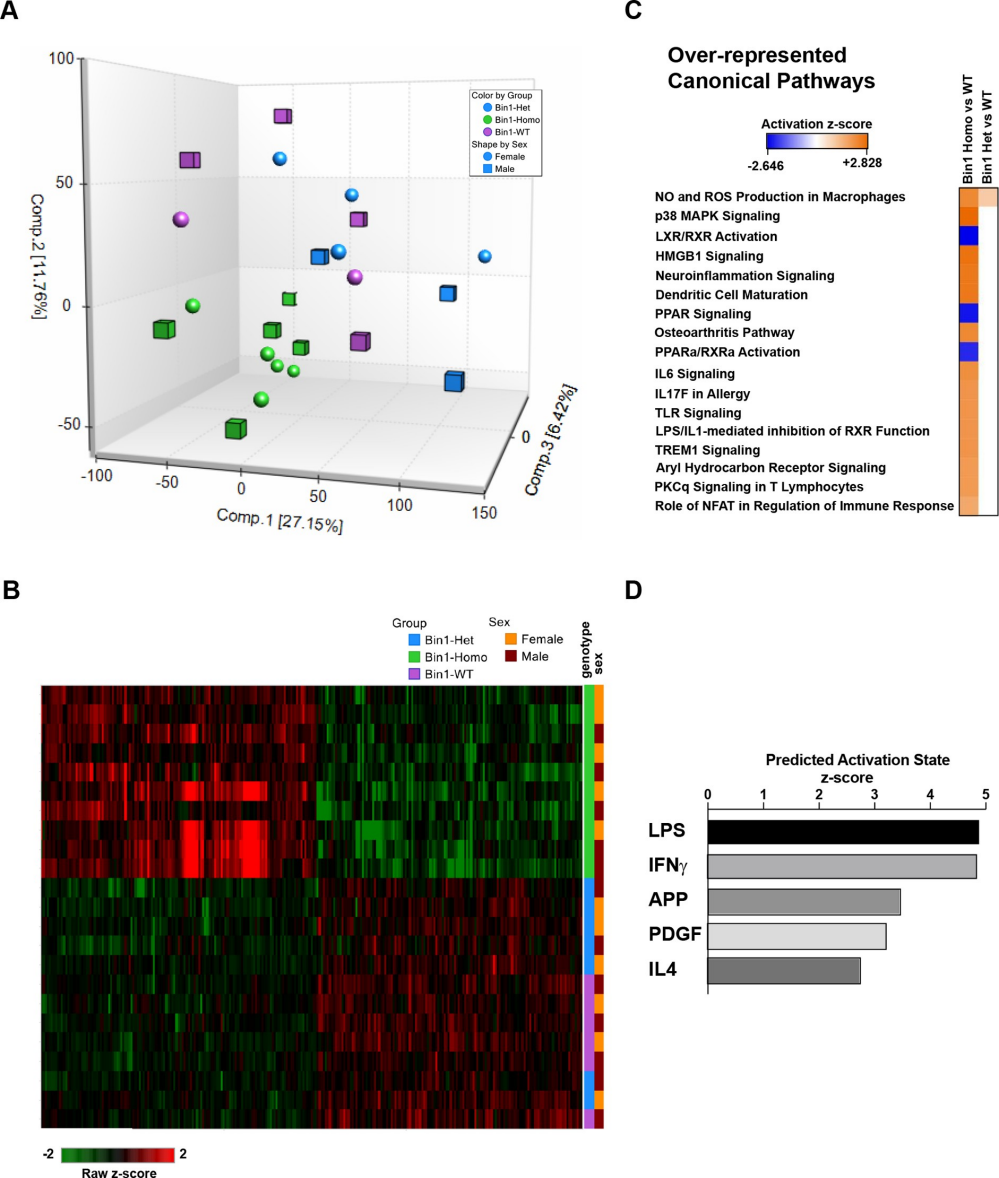
Loss of Bin1 in neurons affects gene expression in microglia *in vivo*. **(A)** Principal component analysis (PCA) representing the tridimensional distribution of the transcriptomes of FACS-sorted microglia from *Bin1*^*flox/flox*^:: *Thyr1-Cre*^*+*^ (green), *Bin1*^*flox/+*^::*Thyr1-Cre*^*+*^ (blue) mice and WT littermates (purple). Cubes indicate males, spheres indicate females. (**B**) Heatmap representing FPKM values of 265 differentially expressed genes (Fold Change |FC| 1.5; padj <0.05) in microglia from *Bin1*^*flox/flox*^:: *Thyr1-Cre*^*+*^ (green), *Bin1*^*flox/+*^::*Thyr1-Cre*^*+*^ (blue) mice and WT littermates (purple), across genotypes and sexes. Brown indicate males, orange indicate females. Green-to-red prism indicates Raw z-score. **(C**) List of the most statistically significant Over-represented Canonical Pathways of the DEGs in microglia from *Bin1*^*flox/flox*^:: *Thyr1-Cre*^*+*^ versus WT littermates and *Bin1*^*flox/+*^::*Thyr1-Cre*^*+*^ versus WT littermates, respectively, obtained by IPA. Blue-to-orange prims indicates Activation z-score. **(D)** List of the most statistically significant Predicted Upstream Activators of the DEGs in microglia from *Bin1*^*flox/flox*^:: *Thyr1-Cre*^*+*^ versus WT littermates, obtained by IPA. Axis represent Predicted activation z-score.

In order to investigate the impact of neuronal dysfunction resulting from Bin1 loss in forebrain excitatory neurons on the surrounding cellular environment, we performed transcriptomic analysis of FACS-sorted microglia from 3 month-old homozygous (*Bin1*^*flox/flox*^:: *Thy1-Cre*^*+*^*)* and heterozygous (*Bin1*^*flox/+*^::*Thy1-Cre*^*+*^*) Bin1-cKO* mice and WT (*Bin1*^*flox/+*^::*Thy1-Cre*^*-*^) littermates. Principal Component Analysis (PCA) showed a clear separation of the transcriptome of microglia from homozygous *Bin1*-*cKO* mice as compared with transcriptomes of microglia from heterozygous *Bin1*-*cKO* and from Bin1 expressing mice, regardless of sex (Fig 5A). Microglia from homozygous *Bin1-cKO* mice showed 265 (|FC| > 1.5, FDR <0.05) statistically significant Differentially Expressed Genes (DEGs) in comparison to WT mice (Fig 5B and S1 Table). Interestingly, microglia from heterozygous *Bin1-cKO* mice showed no statistically significant (FDR <0.05) DEGs in comparison to WT mice (Fig 5B). Of note, loss of neuronal Bin1 didn’t affect microglia number in the hippocampus (S6B and S6C Fig). Upon comparison of gene expression from microglia of homozygous *Bin1*-*cKO* with gene expression from microglia of Bin1 expressing mice by Ingenuity Pathway Analysis (IPA), we observed that the top activated canonical pathway were “NOS and ROS production in Macrophages”, “p38 MAPK Signaling”, “HMGB1 Signaling” and “Neuroinflammation Signaling” (Fig 5C). In addition, the top inhibited canonical pathways were “LXR/RXR Activation”, “PPAR Signaling” and “PPARα/RXRα Activation" (Fig 5C). The top predicted upstream activators were lipo-polysaccharide (LPS), Interferon γ (IFNγ), and Amyloid Precursor Protein (APP) (Fig 5D).

Previously, it had been reported that the conditional expression of the Calpain cleavage product of Cdk5 activator p35 (CK-p25), exclusively in neurons, induces the generation of a mouse model of severe neurodegeneration with AD-like phenotypes[43]. Furthermore, microglia transcriptome from CK-p25 mice showed modules of co-regulated type I and type II interferon response genes[44]. Since we observed a correlation between the transcriptome of microglia homozygous *Bin1*-*cKO* mice with Neuroinflammation Signaling, and in particular IFNγ as predicted upstream activators, we investigated whether we could observe commonalities between the transcriptome of microglia from homozygous *Bin1*-*cKO* mice and the transcriptome of microglia from CK-p25 mice. In the latter, three microglia genes clusters had been identified as relevant: Cluster 3 (491 EnsemblIDs), Cluster 6 (466 EnsemblIDs) and Cluster 7 (233 EnsemblIDs). Our analysis reported an overlap of 18, 27 and 5 genes with the three different clusters, respectively (S2 Table).

CK-p25 mice exhibited increased Aβ levels and aberrant APP processing[43,45]. Furthermore, a recent study reported that transcriptional profiles from CK-p25 mice and 5XFAD (expressing human APP and PSEN1 transgenes with a total of five AD-linked mutations: the Swedish (K670N/M671L), Florida (I716V), and London (V717I) mutations in APP, and the M146L and L286V mutations in PSEN1) mice show similarities [46]. Microglia Single cell RNAseq from 5XFAD mice had been instrumental for the definition of “Disease Associated Microglia” (DAM) gene signature[47]. Since we observed APP as one of the top predicted upstream activators for the DEGs of microglia from *Bin1*^*flox/flox*^:: *Thy1-Cre*^*+*^ mice by IPA, we investigated whether we could observe commonalities between the microglial transcriptome in this study and the DAM signature. Only *Cst7*, *Clec7a*, *Axl* and *Lyz2* out of 40 DAM genes overlapped with the 265 DEGs of microglia from *Bin1*^*flox/flox*^:: *Thy1-Cre*^*+*^ mice. Taken together, such observations suggest that neuronal disfunction resulting from Bin1 loss alters gene expression of the surrounding microglia.

## Discussion

Variation at the *BIN1* locus has shown a consistent, statistically-significant association to LOAD[4,5], although the mechanistic link between BIN1 and AD has remained obscure. In the current study, we focused on the relationship between BIN1 and neuronal loss in the context of Tau pathology[1–3], based on the correlation between AD-associated *BIN1* SNPs and expression levels with Tau deposition[20,28], as well as with various brain morphometric measurements [21], [24][25]. Our analysis showing decreased detection of neuronal Bin1 in the brains of human Tau P301S expressing mice replicates what has been observed in post-mortem brain samples of AD-affected individuals[20],[21], and validate PS19 as a suitable *in vivo* model to understand the contribution of BIN1 to AD-like neuropathology. By employing such a model, we have shown that acute knock-down of total Bin1 in the hippocampus results in a significant neuronal loss. Such observation is consistent with the reported correlation between BIN1 AD-associated SNPs and atrophy of hippocampus, CA1 and parahippocampus, as determined by MRI analysis[21].

Surprisingly, genetic ablation of Bin1 in forebrain excitatory neurons in the context of human Tau P301S expression didn’t affect neuronal number in the hippocampus or cortex. This apparent discrepancy could be explained by the following considerations. First, AAV-mediated Bin1 knock-down completely ablated Bin1 expression (Fig 1C), while Thy1-Cre-mediated Bin1 ablation resulted in a decreased but still detectable levels of neuronal Bin1 (S3A, S3B and S6A Figs). Whether there is a threshold for Bin1 expression that regulates neuronal survival remains to be examined. Second, the AAV employed in the acute knock-down experiment was effective in excitatory and inhibitory neurons as well as astrocytes (S1A and S1B Fig), while Thy1-Cre-mediated Bin1 ablation is specific for forebrain excitatory neurons[34–37]. In light of this observation, we could hypothesize that the decreased but still detectable levels of neuronal Bin1 observed by WB on brain lysates could belong to inhibitory neurons (S3A, S3B and S6A Figs). Indeed, our data suggest that reduction of Bin1 reduces neuronal activity. Reduction of inhibitory neuron activity in the hippocampus has been suggested to result in network hyperexcitability[48], and over time this hyperexcitability can lead to neuropathology[49]. However, whether widespread deletion of Bin1 in hippocampal inhibitory neurons alone results in degeneration of hippocampal excitatory neurons remains untested.

The neuronal-specific Bin1 isoform 1 was detected as a doublet due to phosphorylation, as previously reported in rat as well as in murine brain lysates[15,32]. Interestingly, we noticed the appearance of additional Bin1 immuno-reactive bands (Fig 1A and 1B and S3B Fig) more consistently in mice expressing TauP301S in PS19 background (S3B Fig). It would be possible that additional Bin1 immunoreactive bands represent differentially phosphorylated Bin1 proteins. Recently, it has been reported that phosphorylation of Bin1 at T348 promotes the interaction with Tau *in vitro* and that this leads to a reduced Tau toxicity[32]. Furthermore, it has been shown that phospho-BIN1(T348):BIN1 ratio was increased in post-mortem AD brain samples, suggesting a compensatory mechanism to counteract hyper-phosphorylated Tau toxicity[32]. While we have not demonstrated that any of the extra bands in PS19^Hemi^ mice represents Bin1 T348, the observation of such bands might be the result of a compensatory mechanism in mice overexpressing Tau P301S, similar to that observed in post-mortem AD brain samples.

Nevertheless, the genetic ablation of Bin1 in forebrain excitatory neurons resulted in more rapid animal mortality in the context of human mutant Tau expression and an earlier onset of clinical signs, whereas PS19 mice with hippocampal injection of AAV mBin1-shRNA did not shown a difference in survival or the time required to reach Clinical Score 2. Such observation is consistent with the association of hippocampus predominantly with memory[50]. Conversely, selective deletion of Bin1 in excitatory neurons throughout the forebrain likely impacted a variety of functions, as it is evident by the more rapid onset of clinical symptoms (in the context of human mutant Tau expression) and by the observed deficits in both sensorimotor reflex, and forebrain gating of this reflex, suggesting alterations in both cortical and subcortical circuitry[38]. In addition, deletion of Bin1 in forebrain excitatory neurons led to alterations in microglial transcriptome, which may affect survival.

**Fig 6:**
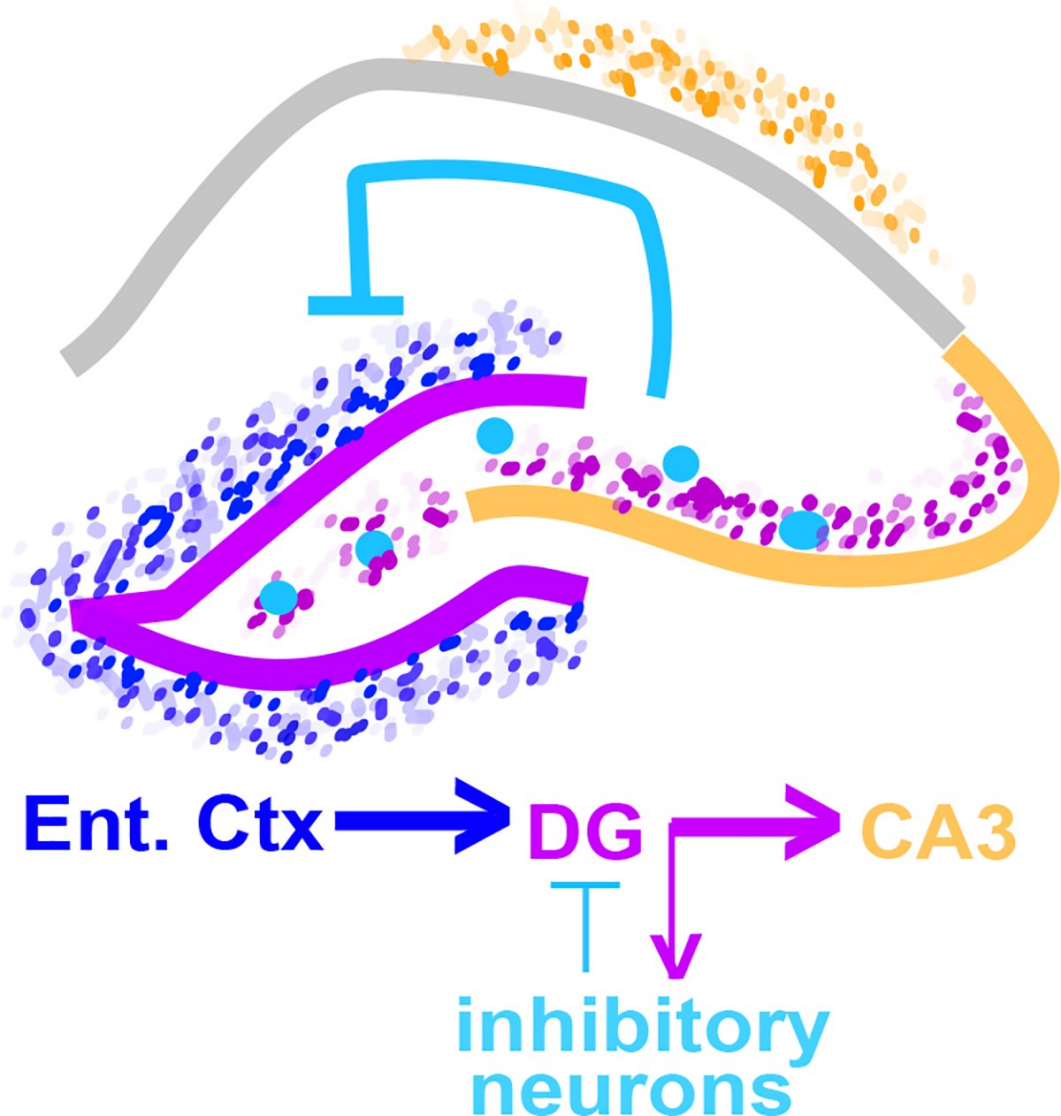
Schematic representation of hippocampal circuitry. Schematic highlighting the potential role for alteration in excitation–inhibition balance to generate differences in DG activation. Entorhinal cortical projections are the main inputs to the DG (shown as dark blue dots). The DG has axonal outputs (shown as purple dots) to CA3 as well as to local inhibitory neurons (large light blue circles). Local inhibitory neurons project back to the DG and inhibit activation.

Neuronal BIN1 has been previously involved in neurite growth, presynaptic cytoskeleton structural integrity, and fission of synaptic vesicles in neurons[11,12,17,19]. Furthermore, mice lacking Amphiphysin I and Bin1 in neurons showed defects in synaptic vesicle recycling, increased mortality, major learning deficits and higher propensity to seizure[11]. In cardiomyocytes, Bin1 is critical for both T-tubule formation, necessary for excitation-contraction coupling, as well as clustering of calcium channels[51], and these functions map to different domains of the Bin1 protein. Recently, Schurmann and colleagues have shown that alterations of Bin1 levels lead to changes in spine morphology, AMPA receptor surface expression and trafficking, and AMPA receptor-mediated synaptic transmission. Bin1 binds Arf6, and may participate the trafficking of vesicular structures through regulation of Arf6 GTPase activity and regulate synaptic AMPA receptor expression[52]. Consistent with these data, we observed that reduction of Bin1 resulted in decreased intracellular calcium release in response to NMDA *in vitro*. Further, the loss of neuronal Bin1 resulted in a reduction of neuronal activation *in vivo* as measured by reduced c-fos-expressing DG granule neurons under low levels of stimulation in the Home Cage environment. This is in contrast to the increased c-fos activation in the DG region in mice exposed to a novel environment. A theoretical model to explain this finding for the DG could involve alterations in excitation–inhibition balance. The DG receives strong feedback inhibition [53] so a decrease in excitatory neuronal activity, potentially due to Bin1 loss, might therefore lead to a subsequent failure of inhibition [53] and hence increased activation in the DG in response to physiological stimuli (Fig 6). In light of the deep complexity of the hippocampal circuitry, and in absence of electrophysiological data for neuronal activity following alteration of Bin1, further experiments are required to substantiate such a model. Although a recent report suggests that BIN1 overexpression promotes the recovery of tauopathy-induced long-term memory deficits[32], loss of Bin1 in forebrain excitatory neurons didn’t alter memory performance in the contextual fear conditioning task, although this may reflect the strength of the training paradigm used here, or compensatory changes in circuitry[54]. Interestingly, network hyperexcitability has been observed in AD-affected individuals[55]. Patients with mild cognitive impairment or dementia due to AD have strong epileptiform activity[55] and such aberrant activity is recapitulated in several rodent models of AD expressing Aβ and hyperphosphorylated Tau[56,57]. Thus, it is plausible that alteration in neuronal BIN1 expression would lead to an unbalanced hippocampal circuitry resulting in memory impairments.

Altered neuronal functionality resulting from the ablation of Bin1 impacted the transcriptome, but not number, of the surrounding microglia. The loss of 2 copies of Bin1 in neurons resulted in differential expression of genes involved in several pro-inflammatory pathways. The presence of IFNγ and APP among the upstream predicted regulators prompted us to investigate the potential overlap between microglia transcriptome from *Bin1 c-KO* mice and microglia transcriptomes from CK-p25 and 5XFAD mice. *Bin1 c-KO* mice didn’t show signs of neuronal loss and microglia proliferation, while CK-p25 and 5XFAD mice showed advert neuronal loss and reactive microglia features [43,45][46]. The only partial overlap between *Bin1 c-KO* and CK-p25/5XFAD microglia transcriptome likely reflects the difference in the severity of the phenotypes observed in the different murine models.

In summary, our data shows that post-natal Bin1 deletion in excitatory forebrain neurons results in moderate but significant decrease in cell-autonomous neuronal excitability, likely exacerbated by the presence of aggregated Tau, coupled with non-cell autonomous microglia activation *in vivo*. Our study suggests that the contribution of genetic variation in BIN1 locus to AD risk might produce moderate effects that could lead to major outcomes with time, consistent with the small effect of common risk variants and with a late onset of Alzheimer’s disease.

## Supporting information

S1 Fig Analysis of GFP expression in AAV-injected PS19^Hemi^ mice. **(A)** Immunofluorescence co-staining of GFP, NeuN (neurons), GFAP (astrocytes), Iba1 (microglia) and Gad67 (inhibitory neurons) in hippocampi of PS19^Hemi^ mice injected with AAV (1/8)-GFP-U6-shRNA scrbl. One representative image per group. Scale bar: 600μm. Arrows mark colocalized staining and x marks non-colocalized staining. Inlet scale bar: 50μm. **(B**) Plot representing the mean**±**SD percentage of GFP^+^ cells co-stained with NeuN, Gad67, GFAP and Iba1, respectively, quantified on 4 animals. **(C)** Immunohistochemistry of GFP in hippocampi of PS19^Hemi^ mice injected with AAV (1/8)-GFP-U6-shRNA scrbl and AAV (1/8)-GFP-U6-shRNA mBin1, respectively. One representative image per group. Scale bar: 600μm. **(D**) Plot representing the percentage of GFP staining (GFP area over total area of the hippocampus) for each animal. Circles represent animals injected with AAV (1/8)-GFP-U6-shRNA scrbl: green filled circles represent ipsilateral hemisphere; blue filled circles represent contralateral hemisphere. Squares represent animals injected with AAV (1/8)-GFP-U6-shRNA mBin1: yellow filled squares represent ipsilateral hemisphere; red filled squares represent contralateral hemisphere. Each circle/square represents one single animal.
(TIF)

S2 Fig Acute knock-down of *Bin1* in the hippocampus of PS19^Hemi^ mice doesn’t alter hippocampal area or overall microglia phenotype. **(A)** Immunohistochemical staining of Iba1 in hippocampi of PS19^Hemi^ mice injected with AAV (1/8)-GFP-U6-shRNA scrbl and AAV (1/8)-GFP- U6-shRNA mBin1, respectively. One representative image per group. Scale bar: 2mm. Inlets scale bar: 100μm. **(B**) Plot representing the Total Area of hippocampus expressed as Log of mm^2^ for each animal. **(C)** Plot representing the Log of the number of microglia cells (based on Iba1 staining) per mm^2^ for each animal. **(D**) Plot representing the Log of the Iba1% staining area normalized over the total area of hippocampus for each animal (+2). **(E**) Plot representing the Log area of microglia dendritic processes in the hippocampus, calculated as area of Iba1 staining normalized by the area of microglia soma for each animal. Circles represent animals injected with AAV (1/8)-GFP- U6-shRNA scrbl: green filled circles represent ipsilateral hemisphere; blue filled circles represent contralateral hemisphere. Squares represent animals injected with AAV (1/8)-GFP- U6-shRNA mBin1: yellow filled squares represent ipsilateral hemisphere; red filled squares represent contralateral hemisphere. Each circle/square represents one single animal. A linear mixed effect model with a 2-way fixed effect model with interaction, a random intercept, and a compound symmetric within-subject correlation structure was employed. P-values were adjusted via the glht single step method. Post-hoc comparisons were analyzed between ipsilateral and contralateral hemispheres within animals, as well as between ipsilateral hemispheres across groups and contralateral hemispheres across groups. Adjusted p-value: * p< 0.05; ** <0.01; *** p<0.001.
(TIF)

S3 Fig Loss of neuronal Bin1 accelerates onset of clinical features in *Bin1 c-Ko; PS19^Hemi^* mice in comparison to Bin1-expressing *PS19^Hemi^* mice. (**A**) Western blotting of brain lysates from *Bin1*^*flox/+*^:: *Thy1-Cre*^*-*^ and *Bin1*^*flox/flox*^::*Thyr1-Cre*^*+*^ mice show Bin1 expression. Each lane represents lysate from one animal. Tubulin represents loading control. Arrows indicated Neuronal Bin1 isoform 1 (top) and Ubiquitous Bin1 isoform 2 (bottom). (**B**) Western blotting of brain lysates from *Bin1*^*flox/+*^:: *Thy1-Cre*^*-*^:: *PS19*^*Hemi*^ and *Bin1*^*flox/flox*^::*Thyr1-Cre*^*+*^:: *PS19*^*Hemi*^ mice show Bin1 expression. Each lane represents lysate from one animal. Tubulin represents loading control. Arrows indicated Neuronal Bin1 isoform 1 (top) and Ubiquitous Bin1 isoform 2 (bottom). (**C**) Male *Bin1*^*flox/flox*^:: *Thy1-Cre*^*+*^:: *PS19*^*Hemi*^ mice (KO, green line) and *Bin1*^*flox/+*^::*Thy1-Cre*^*+*^::*PS19*^*Hemi*^ (HET, red line) show reduced time to reach Clinical Score 2 (CS2) relative to *Bin1*^*flox/+*^::*Thy1-Cre*^*-*^::*PS19*^*Hemi*^ (WT, black line) mice. Mean time to CS2 WT: 287 days, HET: 281 days (p = 0.419), KO: 250 days (p<0.0001) (Kaplan Meier adjusted p-value (adj), relative to WT). (**D**) Female *Bin1*^*flox/flox*^:: *Thy1-Cre*^*+*^:: *PS19*^*Hemi*^ mice (KO, green line) and *Bin1*^*flox/+*^::*Thy1-Cre*^*+*^::*PS19*^*Hemi*^ (HET, red line) show reduced time to reach Clinical Score 2 (CS2) relative to *Bin1*^*flox/+*^::*Thy1-Cre*^*-*^::*PS19*^*Hemi*^ (WT, black line) mice. Mean time to CS2 WT: 349 days, HET: 308 days (p = 0.045), KO: 275 days (p<0.0001) (Kaplan Meier adjusted p-value (adj), relative to WT). (**E**) Male *Bin1*^*flox/flox*^:: *Thy1-Cre*^*+*^:: *PS19*^*Hemi*^ mice (KO, green line) and *Bin1*^*flox/+*^::*Thy1-Cre*^*+*^::*PS19*^*Hemi*^ (HET, red line) show reduced time to reach Clinical Score 3 (CS3) relative to *Bin1*^*flox/+*^::*Thy1-Cre*^*-*^::*PS19*^*Hemi*^ (WT, black line) mice. Mean time to CS2 WT: 308 days, HET: 287 days (p = 0.202), KO: 256 days (p = 0.008) (Kaplan Meier adjusted p-value (adj), relative to WT). (**F**) Female *Bin1*^*flox/flox*^:: *Thy1-Cre*^*+*^:: *PS19*^*Hemi*^ mice (KO, green line) and *Bin1*^*flox/+*^::*Thy1-Cre*^*+*^::*PS19*^*Hemi*^ (HET, red line) show reduced time to reach Clinical Score 3 (CS3) relative to *Bin1*^*flox/+*^::*Thy1-Cre*^*-*^::*PS19*^*Hemi*^ (WT, black line) mice. Mean time to CS2 WT: 363 days, HET: 317 days (p = 0.112), KO: 308 days (p = 0.001) (Kaplan Meier adjusted p-value (adj), relative to WT). Adjusted p-value: * p< 0.05; ** <0.01; *** p<0.001.
(TIF)

S4 Fig Loss of neuronal Bin1 doesn’t affect neuropathological features in *Bin1 c-Ko* mice, regardless the expression of human Tau P301S. Plots representing the Area NeuN staining **(A)** and Total Area of the hippocampus (**B**), expressed in mm^2^, in sections from the hippocampi of male and female *Bin1*^*flox/+*^::*Thy1-Cre*^*-*^::*PS19*^*Ncar*^ (black filled circles) or *Bin1*^*flox/flox*^:: *Thy1-Cre*^*+*^:: *PS19*^*Ncar*^ mice (blue filled circles) mice. Plots representing the Area NeuN staining **(C)** and Total Area of the hippocampus (**D**), expressed in mm^2^, in sections from the hippocampi of male and female *Bin1*^*flox/+*^::*Thy1-Cre*^*-*^::*PS19*^*Hemi*^ (gray filled circles) or *Bin1*^*flox/flox*^:: *Thy1-Cre*^*+*^:: *PS19*^*Hemi*^ mice (purple filled circles) mice. Plots representing the Area of NeuN staining (**E**) and Total Area of the cortex (**F**), expressed in mm^2^, in sections from the cortexes from male and female *Bin1*^*flox/+*^::*Thy1-Cre*^*-*^::*PS19*^*Ncar*^ (black filled circles) or *Bin1*^*flox/flox*^:: *Thy1-Cre*^*+*^:: *PS19*^*Ncar*^ mice (blue filled circles) mice. Plots the Area of NeuN staining (**G**) and Total Area of the cortex (**H**), expressed in mm^2^, in sections from the cortexes from male and female *Bin1*^*flox/+*^::*Thy1-Cre*^*-*^::*PS19*^*Hemi*^ (gray filled circles) or *Bin1*^*flox/flox*^:: *Thy1-Cre*^*+*^:: *PS19*^*Hemi*^ mice (purple filled circles) mice. Each circle represents the value from one animal. 2-way ANOVA with interaction with single-step p-value correction for all endpoints, except Total Area Hippocampus (**B**) 2-way un-equal variance ANOVA with interaction with single-step p-value correction. Results presented in the figure don’t show statistically significant differences.
(TIF)

S5 Fig Loss of neuronal Bin1 mildly affects behavior in *Bin1 c-Ko* mice. **(A)** Plot representing the Total Distance traveled (mm) by mice at 5 and 6 months of age. *Bin1*^*flox/flox*^:: *Thy1-Cre*^*-*^::*PS19*^*Ncar*^ N = 16 (5 month-old), 24 (6 month-old) (black filled circles) or *Bin1*^*flox/flox*^:: *Thy1-Cre*^*+*^:: *PS19*^*Ncar*^ mice N = 18 (5 month-old), 24 (6 month-old) (blue filled circles) mice. *Bin1*^*flox/flox*^:: *Thy1-Cre*^*-*^::*PS19*^*Hemi*^ N = 10 (5 month-old), 13 (6 month-old) (gray filled circles) or *Bin1*^*flox/flox*^:: *Thy1-Cre*^*+*^:: *PS19*^*Hemi*^ mice N = 12 (5 month-old), 20 (6 month-old) (purple filled circles) mice. Two-way ANOVA with Tukey’s multiple comparison test. Main measures for each time point: genotype, time, interaction: genotype and time, followed by post-hoc comparisons to *Bin1*^*flox/flox*^:: *Thy1-Cre*^*-*^::*PS19*^*Ncar*^ mice. *Bin1*^*flox/flox*^:: *Thy1-Cre*^*+*^:: *PS19*^*Ncar*^ mice show significantly reduced open field exploration at 6m. p-value: * p< 0.05; ** <0.01; *** p<0.001. (**B**) Plot representing the Startle Amplitude, expressed as arbitrary units, of mice at 6 and 7 months of age. *Bin1*^*flox/flox*^:: *Thy1-Cre*^*-*^::*PS19*^*Ncar*^ N = 24 (6 month-old), 23 (7 month-old) (black filled circles) or *Bin1*^*flox/flox*^:: *Thy1-Cre*^*+*^:: *PS19*^*Ncar*^ mice N = 23 (6 month-old), 23 (7 month-old) (blue filled circles) mice. *Bin1*^*flox/flox*^:: *Thy1-Cre*^*-*^::*PS19*^*Hemi*^ N = 15 (6 month-old), 15 (7 month-old) (gray filled circles) or *Bin1*^*flox/flox*^:: *Thy1-Cre*^*+*^:: *PS19*^*Hemi*^ mice N = 19 (6 month-old), 18 (7 month-old) (purple filled circles) mice. Two-way ANOVA with Tukey’s multiple comparison test, main measures for each time point: genotype, time, interaction: genotype and time, followed by post-hoc comparisons to *Bin1*^*flox/flox*^:: *Thy1-Cre*^*-*^::*PS19*^*Ncar*^ mice. *Bin1*^*flox/flox*^:: *Thy1-Cre*^*+*^:: *PS19*^*Ncar*^ and *Bin1*^*flox/flox*^:: *Thy1-Cre*^*+*^:: *PS19*^*Hemi*^ genotypes show reduced auditory startle. p-value: * p< 0.05; ** <0.01; *** p<0.001. (**C**) Plot representing the percentage Pre-Pulse Inhibition (PPI) of mice at 6 and 7 months of age. *Bin1*^*flox/flox*^:: *Thy1-Cre*^*-*^::*PS19*^*Ncar*^ N = 24 (6 month-old), 23 (7 month-old) (black filled circles) or *Bin1*^*flox/flox*^:: *Thy1-Cre*^*+*^:: *PS19*^*Ncar*^ mice N = 23 (6 month-old), 23 (7 month-old) (blue filled circles) mice. *Bin1*^*flox/flox*^:: *Thy1-Cre*^*-*^::*PS19*^*Hemi*^ N = 15 (6 month-old), 15 (7 month-old) (gray filled circles) or *Bin1*^*flox/flox*^:: *Thy1-Cre*^*+*^:: *PS19*^*Hemi*^ mice N = 19 (6 month-old), 18 (7 month-old) (purple filled circles) mice. Two-way ANOVA with Tukey’s multiple comparison test, main measures for each time point: genotype, time, interaction: genotype and time, followed by post-hoc comparisons to *Bin1*^*flox/flox*^:: *Thy1-Cre*^*-*^::*PS19*^*Ncar*^ mice. *Bin1*^*flox/flox*^:: *Thy1-Cre*^*+*^:: *PS19*^*Hemi*^ genotype mice show reduced Pre-Pulse Inhibition (PPI). p-value: * p< 0.05; ** <0.01; *** p<0.001. (**D**) Plots representing the percentage of freezing time for male (left plot) and female (right plot) mice during Contextual Fear Conditioning. Two-way ANOVA with Tukey’s multiple comparison test. (**E**) Plots representing the percentage of freezing time for male (left plot) and female (right plot) mice during Cued Fear Conditioning. *Bin1*^*flox/+*^:: *Thy1-Cre*^*-*^::*PS19*^*Ncar*^ (black filled circles) or *Bin1*^*flox/flox*^:: *Thy1-Cre*^*+*^:: *PS19*^*Ncar*^ mice (blue filled circles) mice. *Bin1*^*flox/+*^:: *Thy1-Cre*^*-*^::*PS19*^*Hemi*^ (gray filled circles) or *Bin1*^*flox/flox*^:: *Thy1-Cre*^*+*^:: *PS19*^*Hemi*^ mice (purple filled circles) mice. Each circle represents the value from one animal. Two-way ANOVA with Tukey’s multiple comparison test. Results presented in D and E don’t show statistically significant differences.
(TIF)

S6 Fig Loss of neuronal Bin1 doesn’t affect microglia number in *Bin1 c-Ko* mice. (**A**) Western blotting of brain lysates from *Bin1*^*flox/+*^:: *Thy1-Cre*^*-*,^ *Bin1*^*flox/+*^::*Thy1-Cre*^*+*^ and *Bin1*^*flox/flox*^::*Thyr1-Cre*^*+*^ mice showing Bin1 expression. Each lane represents lysate from one animal. Gapdh represents loading control. Arrows indicated Neuronal Bin1 isoform 1 (top) and Ubiquitous Bin1 isoform 2 (bottom). (**B**) Representative immunofluorescence images of Iba1 labeling in select brain regions from *Bin1*^*flox/+*^:: *Thy1-Cre*^*-*^ and *Bin1*^*flox/flox*^:: *Thy1-Cre*^*+*^ mice. Scale bar: 300μm. Inlets scale bar: 50μm. (**C**) Plot representing the number of Iba1^+^ cells per image. *Bin1*^*flox/+*^:: *Thy1-Cre*^*-*^ (black bars) and *Bin1*^*flox/flox*^:: *Thy1-Cre*^*+*^ (blue bars). Boxes represent range, with line representing mean. N = 3 per group. Two-way ANOVA with Tukey’s multiple comparison test. Results presented in C don’t show statistically significant differences.
(TIF)

S7 Fig Full Western Blots representing images used in Fig 1A, 1B and 1C. (TIF)

S8 Fig Full Western Blots representing images used in Fig 4A. (TIF)

S9 Fig Full Western Blots representing images used in S3A and S3B Fig. (TIF)

S10 Fig Full Western Blots representing images used in S6A Fig. (TIF)

S1 Table Microglia gene expression analysis. RNAseq gene expression analysis of microglia from *Bin1*^*flox/flox*^:: *Thy1-Cre*^*+*^, *Bin1*^*flox/+*^:: *Thy1-Cre 1-Cre*^*+*^ and *Bin1*^*flox/+*^:: *Thy1-Cre 1-Cre*^*-*^ males and females mice at 3 months of age.
(XLSX)

S2 Table Overlap between microglia DEGs from *Bin1 c-Ko* and microglia DEGs from CK-p25 mice. List of differentially expressed genes commonly shared by transcriptome of microglia from *Bin1*^*flox/flox*^:: *Thy1-Cre*^*+*^ and the transcriptome of microglia from CK-p25 mice clusters 3, 6 and 7, respectively.
(XLSX)
