# Supplementary figures and images for: Cell-autonomous and non-cell autonomous effects of neuronal BIN1 loss in vivo
Source: PLoS One. 2019 Aug 13;14(8):e0220125. doi: 10.1371/journal.pone.0220125 (PMC6692034; doi:10.1371/journal.pone.0220125)

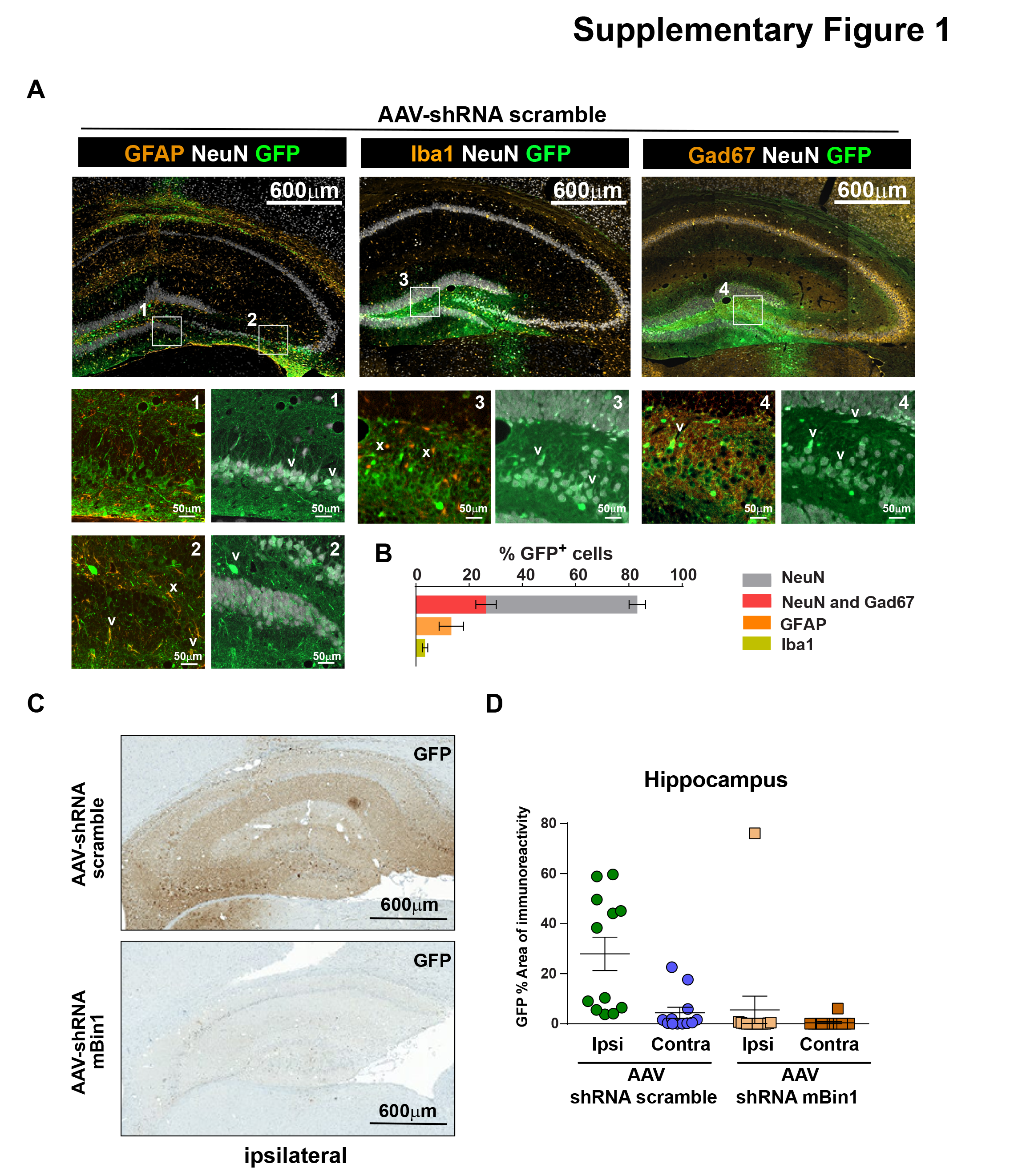

Supplement: S1 Fig — (A) Immunofluorescence co-staining of GFP, NeuN (neurons), GFAP (astrocytes), Iba1 (microglia) and Gad67 (inhibitory neurons) in hippocampi of PS19Hemi mice injected with AAV (1/8)-GFP-U6-shRNA scrbl. One representative image per group. Scale bar: 600μm. Arrows mark colocalized staining and x marks non-colocalized staining. Inlet scale bar: 50μm. (B) Plot representing the mean±SD percentage of GFP+ cells co-stained with NeuN, Gad67, GFAP and Iba1, respectively, quantified on 4 animals. (C) Immunohistochemistry of GFP in hippocampi of PS19Hemi mice injected with AAV (1/8)-GFP-U6-shRNA scrbl and AAV (1/8)-GFP-U6-shRNA mBin1, respectively. One representative image per group. Scale bar: 600μm. (D) Plot representing the percentage of GFP staining (GFP area over total area of the hippocampus) for each animal. Circles represent animals injected with AAV (1/8)-GFP-U6-shRNA scrbl: green filled circles represent ipsilateral hemisphere; blue filled circles represent contralateral hemisphere. Squares represent animals injected with AAV (1/8)-GFP-U6-shRNA mBin1: yellow filled squares represent ipsilateral hemisphere; red filled squares represent contralateral hemisphere. Each circle/square represents one single animal. (TIF) [file pone.0220125.s001.tif]

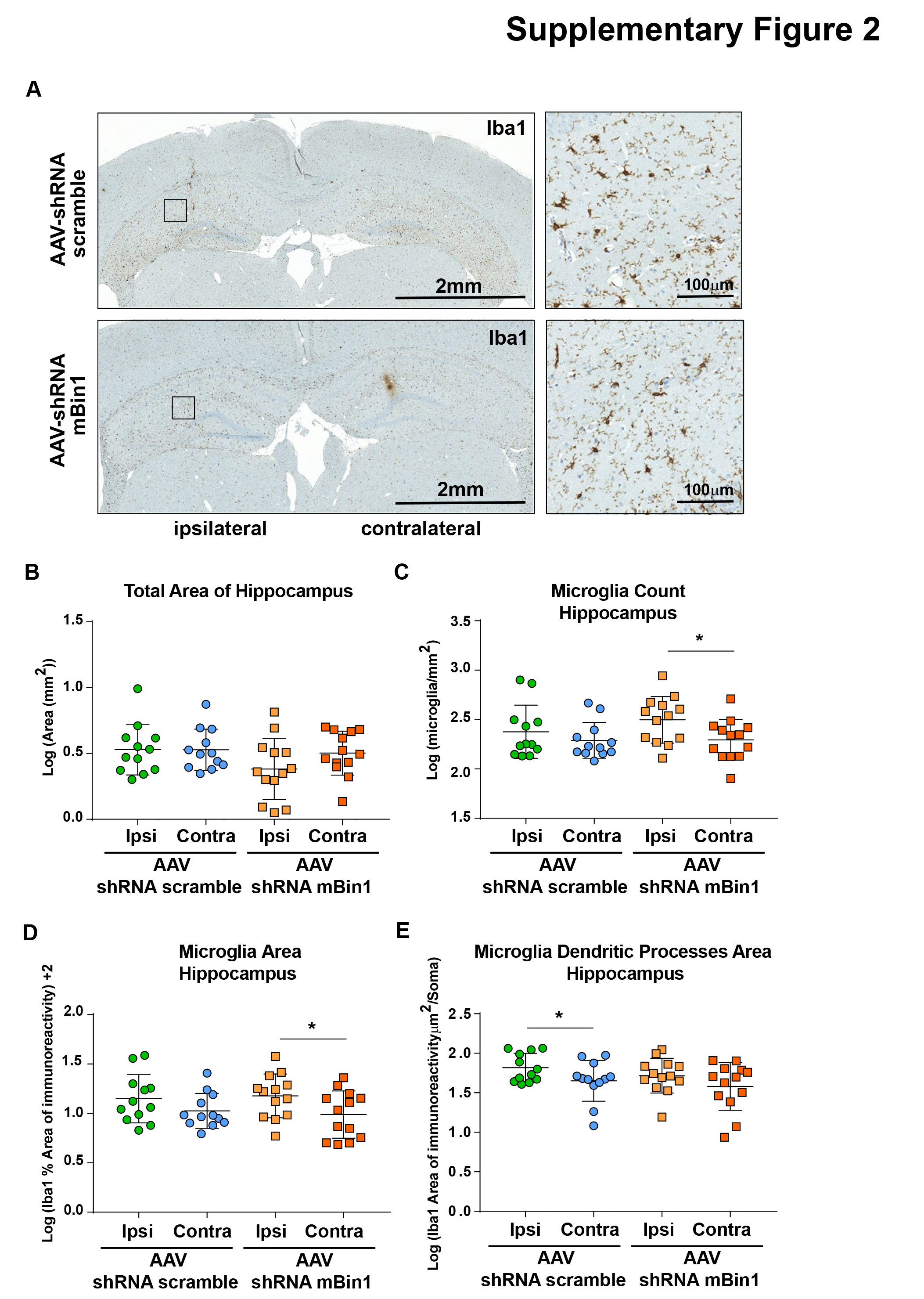

Supplement: S2 Fig — (A) Immunohistochemical staining of Iba1 in hippocampi of PS19Hemi mice injected with AAV (1/8)-GFP-U6-shRNA scrbl and AAV (1/8)-GFP- U6-shRNA mBin1, respectively. One representative image per group. Scale bar: 2mm. Inlets scale bar: 100μm. (B) Plot representing the Total Area of hippocampus expressed as Log of mm2 for each animal. (C) Plot representing the Log of the number of microglia cells (based on Iba1 staining) per mm2 for each animal. (D) Plot representing the Log of the Iba1% staining area normalized over the total area of hippocampus for each animal (+2). (E) Plot representing the Log area of microglia dendritic processes in the hippocampus, calculated as area of Iba1 staining normalized by the area of microglia soma for each animal. Circles represent animals injected with AAV (1/8)-GFP- U6-shRNA scrbl: green filled circles represent ipsilateral hemisphere; blue filled circles represent contralateral hemisphere. Squares represent animals injected with AAV (1/8)-GFP- U6-shRNA mBin1: yellow filled squares represent ipsilateral hemisphere; red filled squares represent contralateral hemisphere. Each circle/square represents one single animal. A linear mixed effect model with a 2-way fixed effect model with interaction, a random intercept, and a compound symmetric within-subject correlation structure was employed. P-values were adjusted via the glht single step method. Post-hoc comparisons were analyzed between ipsilateral and contralateral hemispheres within animals, as well as between ipsilateral hemispheres across groups and contralateral hemispheres across groups. Adjusted p-value: * p< 0.05; ** <0.01; *** p<0.001. (TIF) [file pone.0220125.s002.tif]

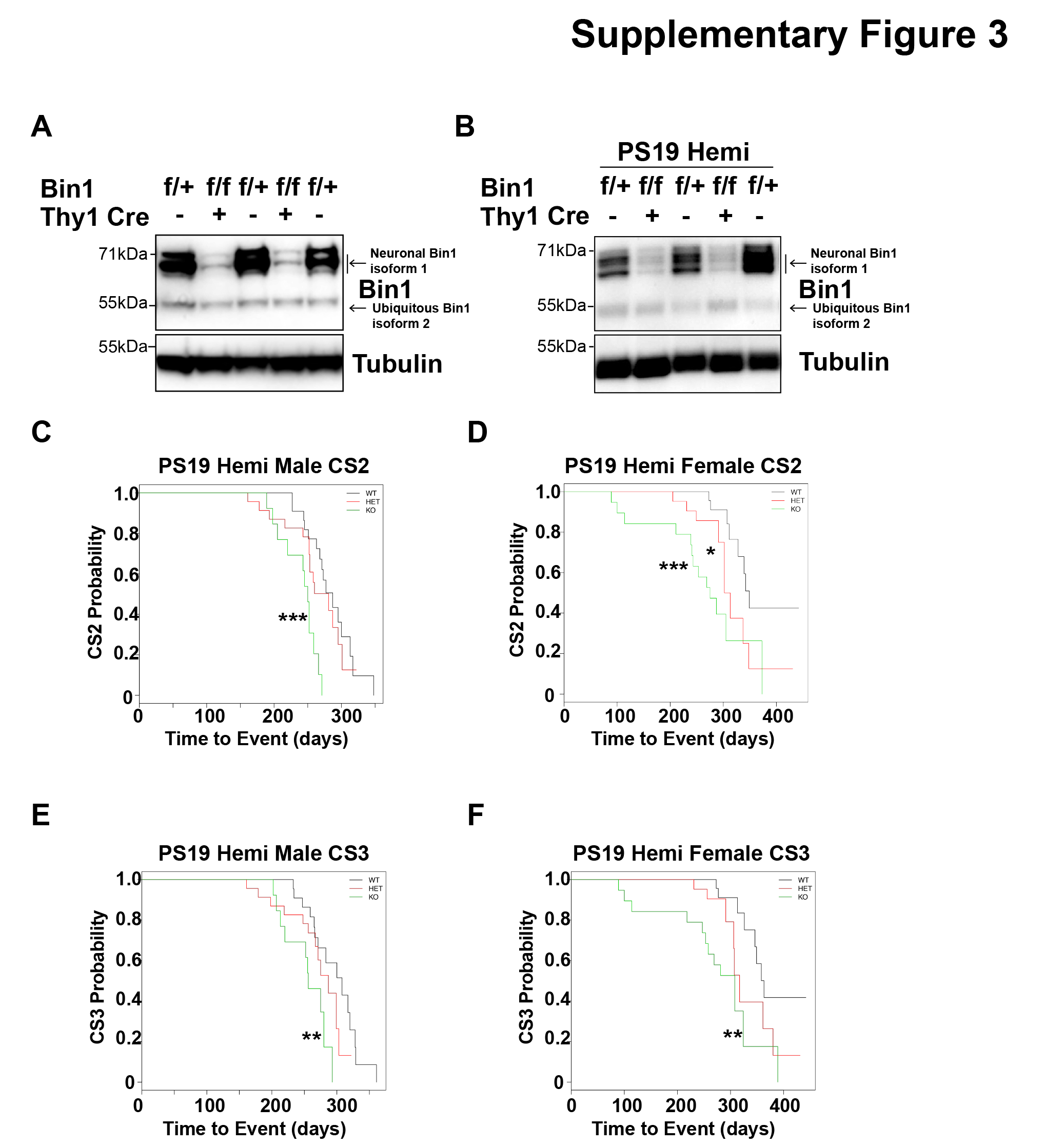

Supplement: S3 Fig — (A) Western blotting of brain lysates from Bin1flox/+:: Thy1-Cre- and Bin1flox/flox::Thyr1-Cre+ mice show Bin1 expression. Each lane represents lysate from one animal. Tubulin represents loading control. Arrows indicated Neuronal Bin1 isoform 1 (top) and Ubiquitous Bin1 isoform 2 (bottom). (B) Western blotting of brain lysates from Bin1flox/+:: Thy1-Cre-:: PS19Hemi and Bin1flox/flox::Thyr1-Cre+:: PS19Hemi mice show Bin1 expression. Each lane represents lysate from one animal. Tubulin represents loading control. Arrows indicated Neuronal Bin1 isoform 1 (top) and Ubiquitous Bin1 isoform 2 (bottom). (C) Male Bin1flox/flox:: Thy1-Cre+:: PS19Hemi mice (KO, green line) and Bin1flox/+::Thy1-Cre+::PS19Hemi (HET, red line) show reduced time to reach Clinical Score 2 (CS2) relative to Bin1flox/+::Thy1-Cre-::PS19Hemi (WT, black line) mice. Mean time to CS2 WT: 287 days, HET: 281 days (p = 0.419), KO: 250 days (p<0.0001) (Kaplan Meier adjusted p-value (adj), relative to WT). (D) Female Bin1flox/flox:: Thy1-Cre+:: PS19Hemi mice (KO, green line) and Bin1flox/+::Thy1-Cre+::PS19Hemi (HET, red line) show reduced time to reach Clinical Score 2 (CS2) relative to Bin1flox/+::Thy1-Cre-::PS19Hemi (WT, black line) mice. Mean time to CS2 WT: 349 days, HET: 308 days (p = 0.045), KO: 275 days (p<0.0001) (Kaplan Meier adjusted p-value (adj), relative to WT). (E) Male Bin1flox/flox:: Thy1-Cre+:: PS19Hemi mice (KO, green line) and Bin1flox/+::Thy1-Cre+::PS19Hemi (HET, red line) show reduced time to reach Clinical Score 3 (CS3) relative to Bin1flox/+::Thy1-Cre-::PS19Hemi (WT, black line) mice. Mean time to CS2 WT: 308 days, HET: 287 days (p = 0.202), KO: 256 days (p = 0.008) (Kaplan Meier adjusted p-value (adj), relative to WT). (F) Female Bin1flox/flox:: Thy1-Cre+:: PS19Hemi mice (KO, green line) and Bin1flox/+::Thy1-Cre+::PS19Hemi (HET, red line) show reduced time to reach Clinical Score 3 (CS3) relative to Bin1flox/+::Thy1-Cre-::PS19Hemi (WT, black line) mice. Mean time to CS2 WT: 363 days, H [file pone.0220125.s003.tif]

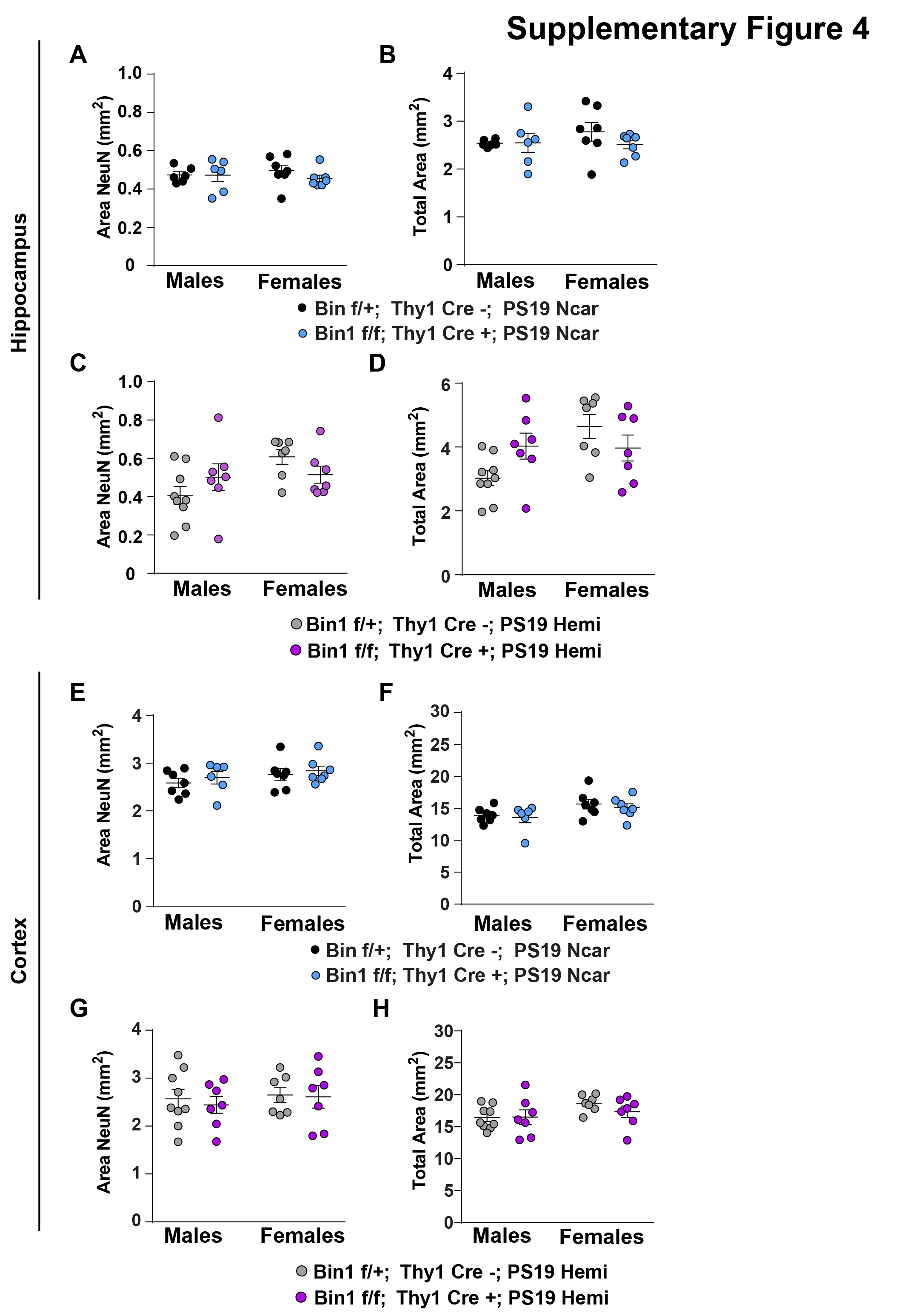

Supplement: S4 Fig — Plots representing the Area NeuN staining (A) and Total Area of the hippocampus (B), expressed in mm2, in sections from the hippocampi of male and female Bin1flox/+::Thy1-Cre-::PS19Ncar (black filled circles) or Bin1flox/flox:: Thy1-Cre+:: PS19Ncar mice (blue filled circles) mice. Plots representing the Area NeuN staining (C) and Total Area of the hippocampus (D), expressed in mm2, in sections from the hippocampi of male and female Bin1flox/+::Thy1-Cre-::PS19Hemi (gray filled circles) or Bin1flox/flox:: Thy1-Cre+:: PS19Hemi mice (purple filled circles) mice. Plots representing the Area of NeuN staining (E) and Total Area of the cortex (F), expressed in mm2, in sections from the cortexes from male and female Bin1flox/+::Thy1-Cre-::PS19Ncar (black filled circles) or Bin1flox/flox:: Thy1-Cre+:: PS19Ncar mice (blue filled circles) mice. Plots the Area of NeuN staining (G) and Total Area of the cortex (H), expressed in mm2, in sections from the cortexes from male and female Bin1flox/+::Thy1-Cre-::PS19Hemi (gray filled circles) or Bin1flox/flox:: Thy1-Cre+:: PS19Hemi mice (purple filled circles) mice. Each circle represents the value from one animal. 2-way ANOVA with interaction with single-step p-value correction for all endpoints, except Total Area Hippocampus (B) 2-way un-equal variance ANOVA with interaction with single-step p-value correction. Results presented in the figure don’t show statistically significant differences. (TIF) [file pone.0220125.s004.tif]

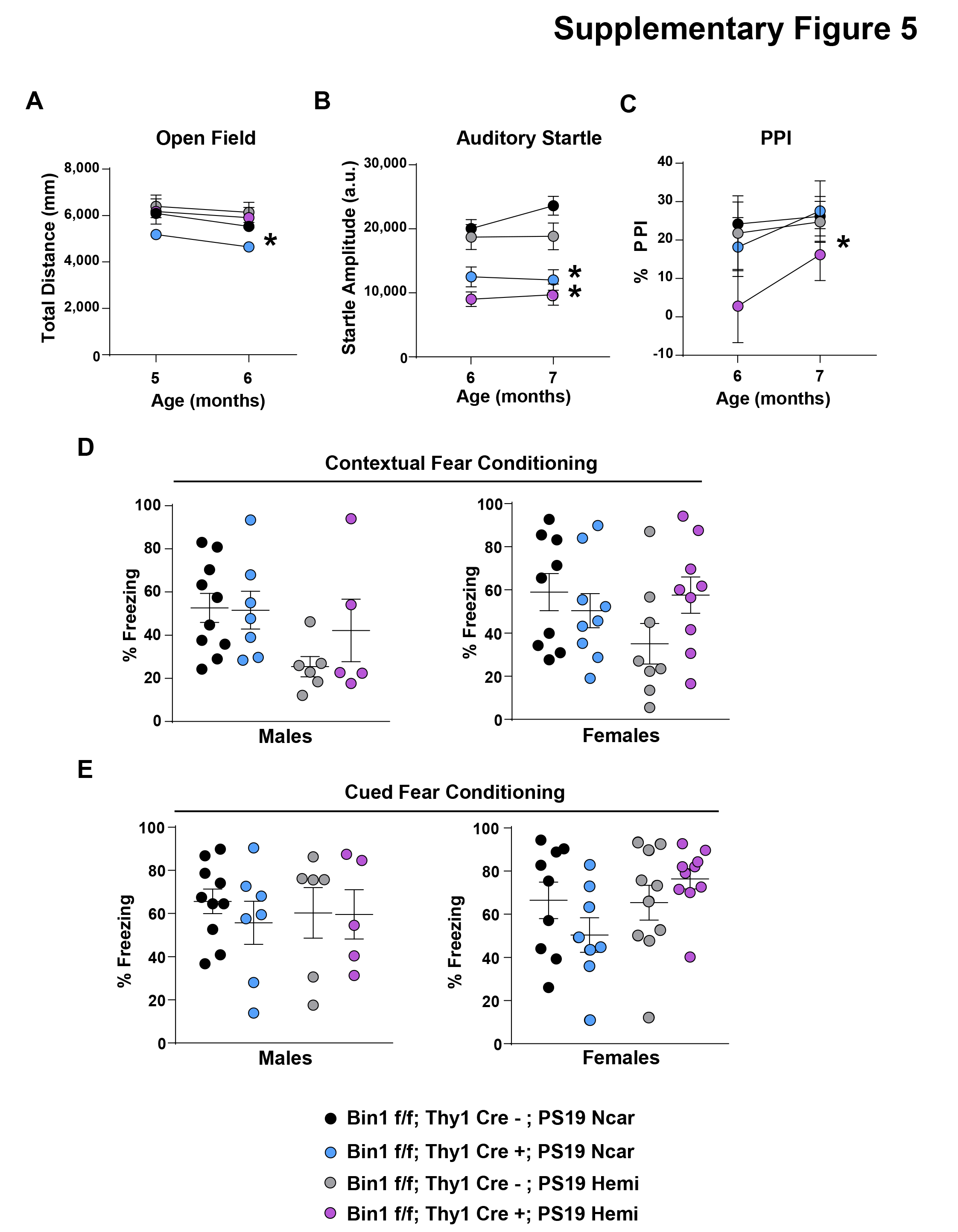

Supplement: S5 Fig — (A) Plot representing the Total Distance traveled (mm) by mice at 5 and 6 months of age. Bin1flox/flox:: Thy1-Cre-::PS19Ncar N = 16 (5 month-old), 24 (6 month-old) (black filled circles) or Bin1flox/flox:: Thy1-Cre+:: PS19Ncar mice N = 18 (5 month-old), 24 (6 month-old) (blue filled circles) mice. Bin1flox/flox:: Thy1-Cre-::PS19Hemi N = 10 (5 month-old), 13 (6 month-old) (gray filled circles) or Bin1flox/flox:: Thy1-Cre+:: PS19Hemi mice N = 12 (5 month-old), 20 (6 month-old) (purple filled circles) mice. Two-way ANOVA with Tukey’s multiple comparison test. Main measures for each time point: genotype, time, interaction: genotype and time, followed by post-hoc comparisons to Bin1flox/flox:: Thy1-Cre-::PS19Ncar mice. Bin1flox/flox:: Thy1-Cre+:: PS19Ncar mice show significantly reduced open field exploration at 6m. p-value: * p< 0.05; ** <0.01; *** p<0.001. (B) Plot representing the Startle Amplitude, expressed as arbitrary units, of mice at 6 and 7 months of age. Bin1flox/flox:: Thy1-Cre-::PS19Ncar N = 24 (6 month-old), 23 (7 month-old) (black filled circles) or Bin1flox/flox:: Thy1-Cre+:: PS19Ncar mice N = 23 (6 month-old), 23 (7 month-old) (blue filled circles) mice. Bin1flox/flox:: Thy1-Cre-::PS19Hemi N = 15 (6 month-old), 15 (7 month-old) (gray filled circles) or Bin1flox/flox:: Thy1-Cre+:: PS19Hemi mice N = 19 (6 month-old), 18 (7 month-old) (purple filled circles) mice. Two-way ANOVA with Tukey’s multiple comparison test, main measures for each time point: genotype, time, interaction: genotype and time, followed by post-hoc comparisons to Bin1flox/flox:: Thy1-Cre-::PS19Ncar mice. Bin1flox/flox:: Thy1-Cre+:: PS19Ncar and Bin1flox/flox:: Thy1-Cre+:: PS19Hemi genotypes show reduced auditory startle. p-value: * p< 0.05; ** <0.01; *** p<0.001. (C) Plot representing the percentage Pre-Pulse Inhibition (PPI) of mice at 6 and 7 months of age. Bin1flox/flox:: Thy1-Cre-::PS19Ncar N = 24 (6 month-old), 23 (7 month-old) (black filled circles) or Bin1flox/flox:: Thy1-Cre+:: P [file pone.0220125.s005.tif]

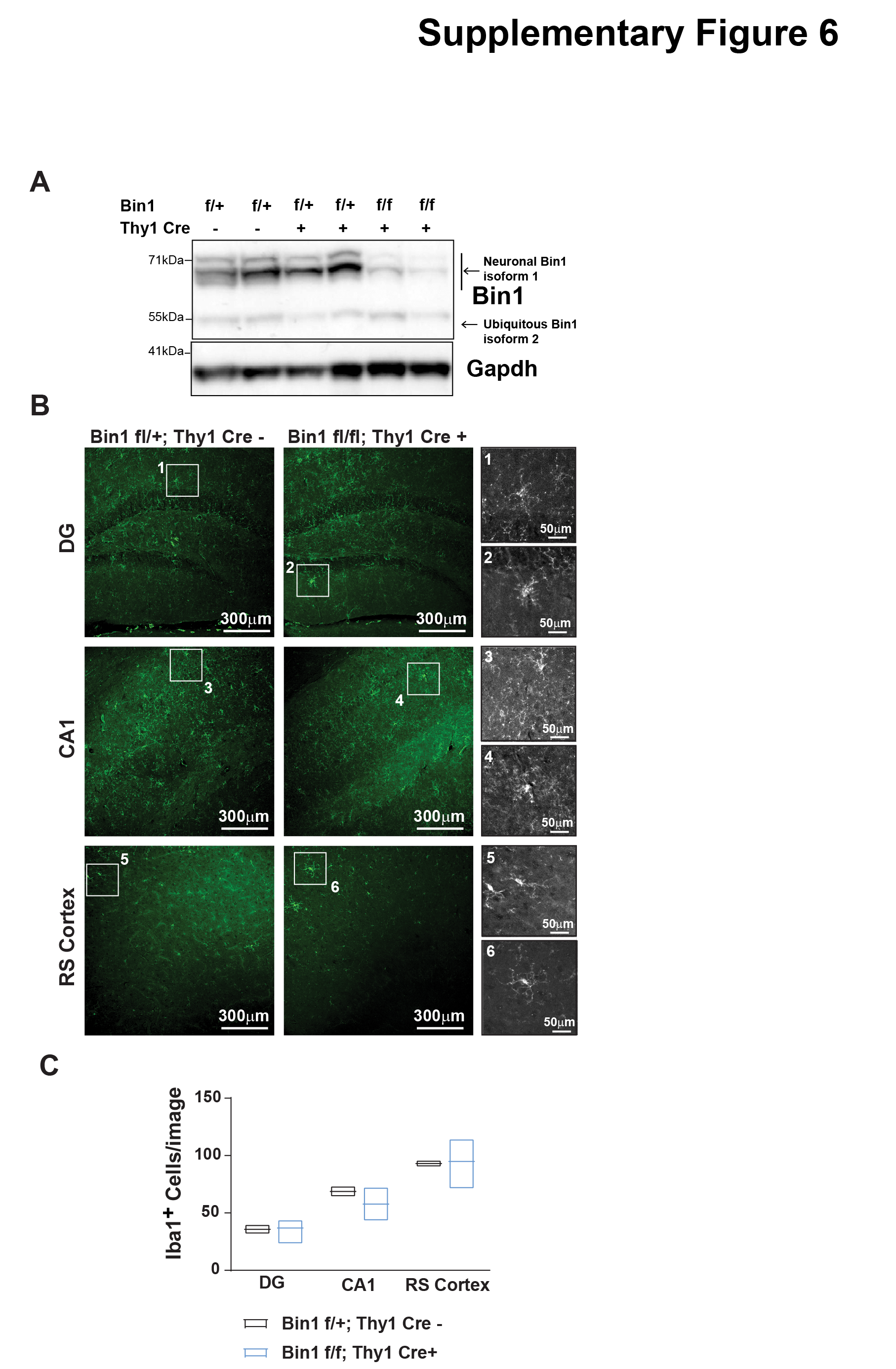

Supplement: S6 Fig — (A) Western blotting of brain lysates from Bin1flox/+:: Thy1-Cre-, Bin1flox/+::Thy1-Cre+ and Bin1flox/flox::Thyr1-Cre+ mice showing Bin1 expression. Each lane represents lysate from one animal. Gapdh represents loading control. Arrows indicated Neuronal Bin1 isoform 1 (top) and Ubiquitous Bin1 isoform 2 (bottom). (B) Representative immunofluorescence images of Iba1 labeling in select brain regions from Bin1flox/+:: Thy1-Cre- and Bin1flox/flox:: Thy1-Cre+ mice. Scale bar: 300μm. Inlets scale bar: 50μm. (C) Plot representing the number of Iba1+ cells per image. Bin1flox/+:: Thy1-Cre- (black bars) and Bin1flox/flox:: Thy1-Cre+ (blue bars). Boxes represent range, with line representing mean. N = 3 per group. Two-way ANOVA with Tukey’s multiple comparison test. Results presented in C don’t show statistically significant differences. (TIF) [file pone.0220125.s006.tif]

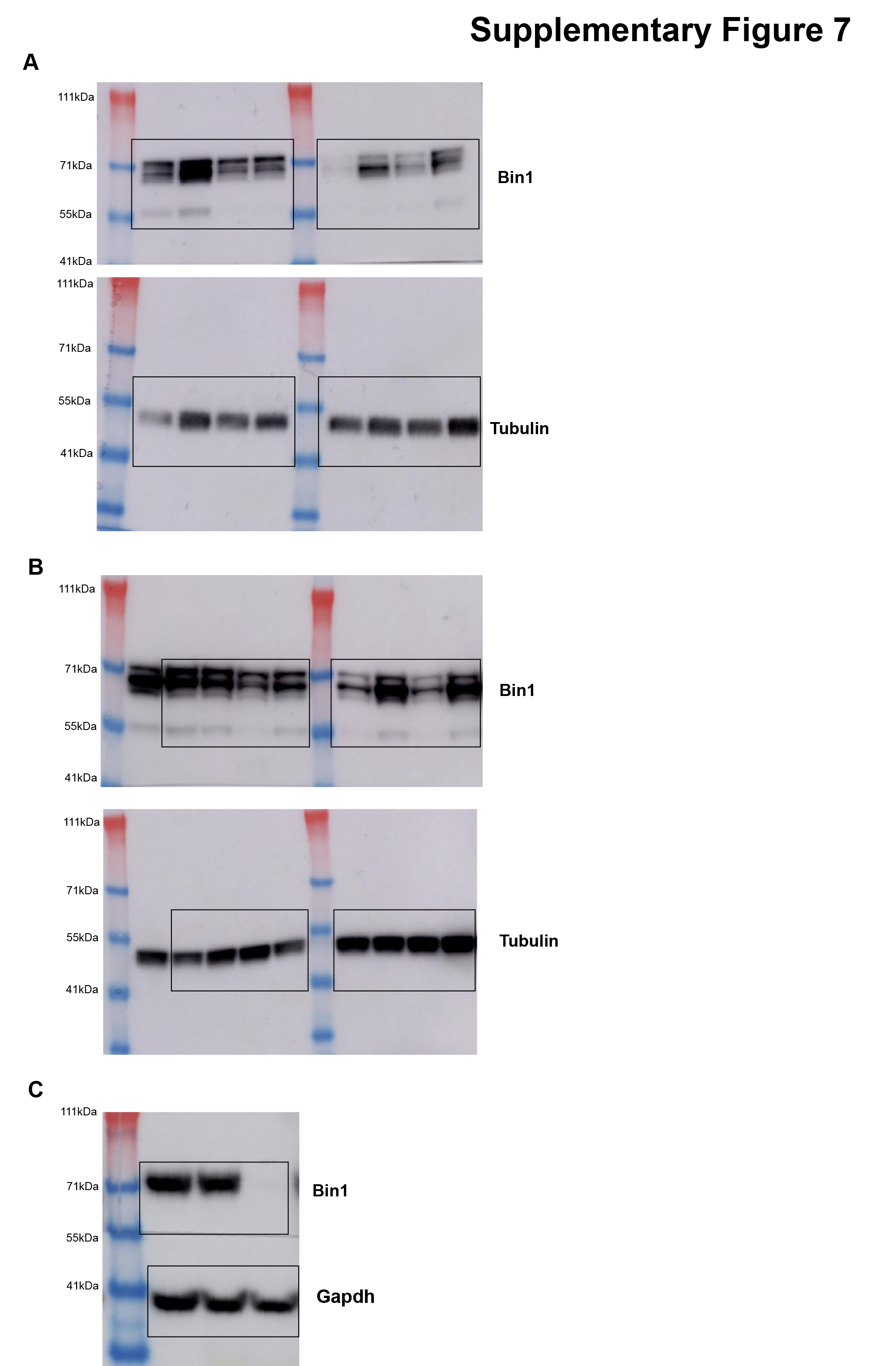

Supplement: S7 Fig — (TIF) [file pone.0220125.s007.tif]

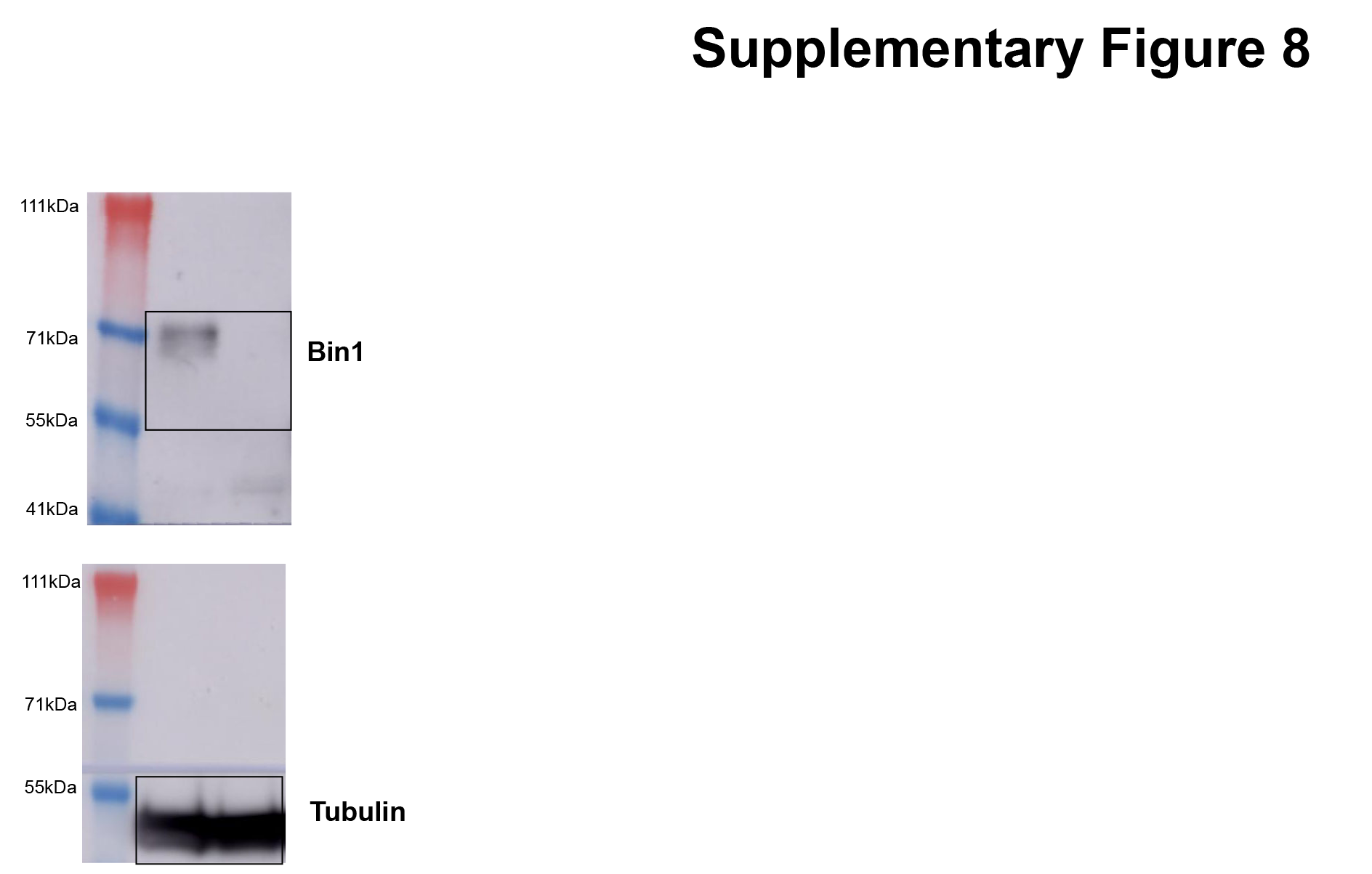

Supplement: S8 Fig — (TIF) [file pone.0220125.s008.tif]

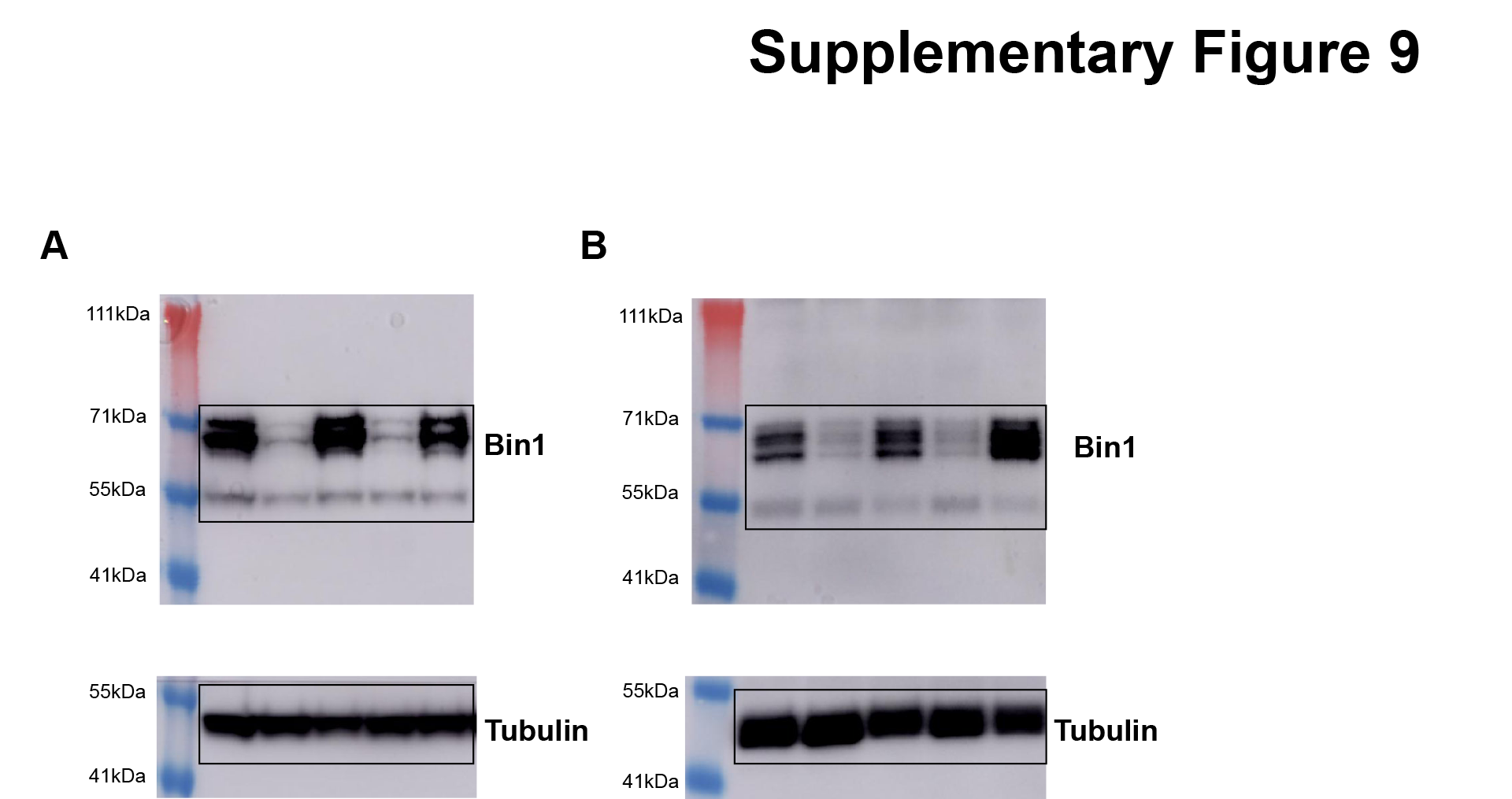

Supplement: S9 Fig — (TIF) [file pone.0220125.s009.tif]

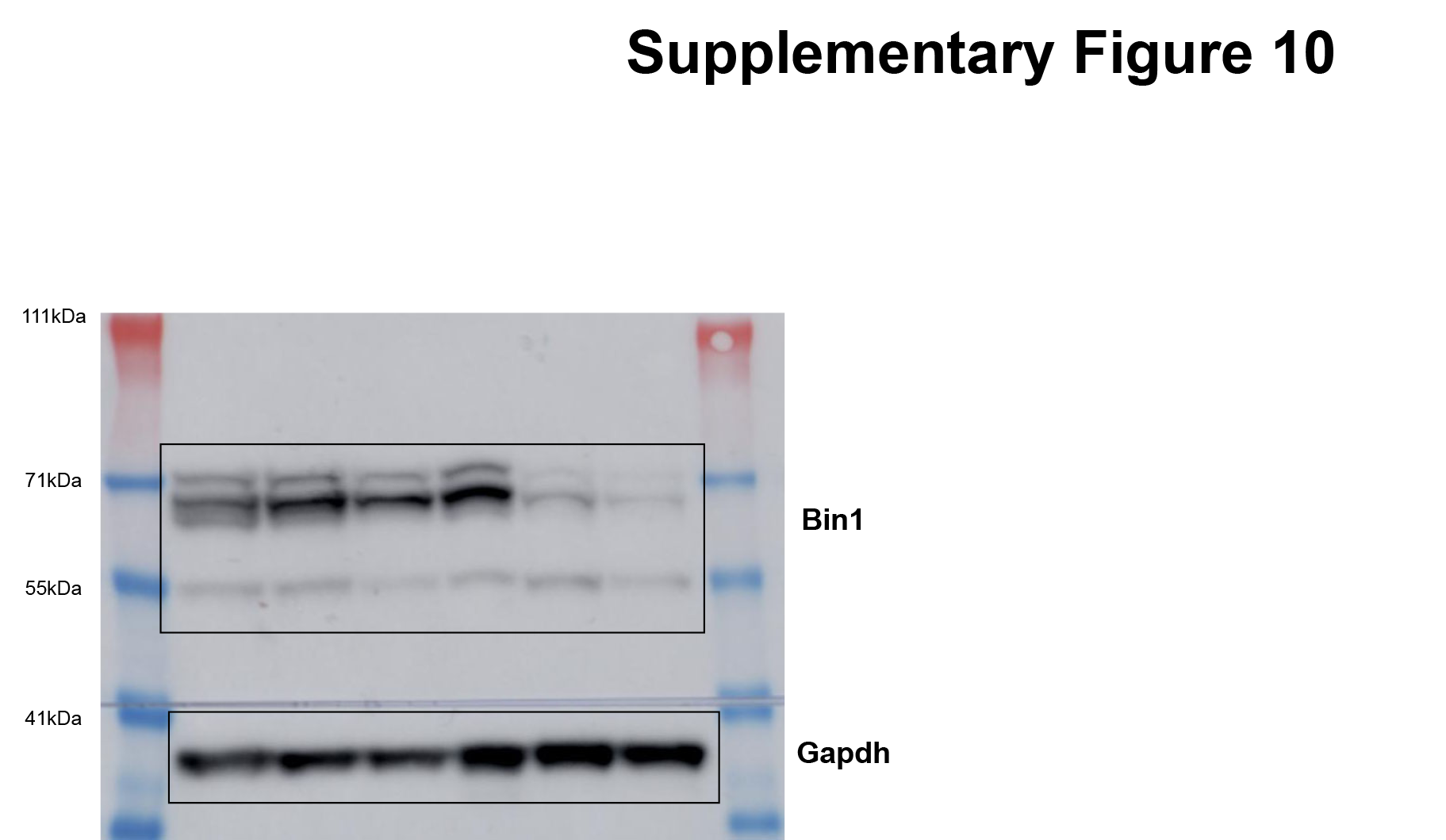

Supplement: S10 Fig — (TIF) [file pone.0220125.s010.tif]
